# Competition for water induced by transnational land acquisitions for agriculture

**DOI:** 10.1038/s41467-022-28077-2

**Published:** 2022-01-26

**Authors:** Davide Danilo Chiarelli, Paolo D’Odorico, Marc F. Müller, Nathaniel D. Mueller, Kyle Frankel Davis, Jampel Dell’Angelo, Gopal Penny, Maria Cristina Rulli

**Affiliations:** 1 Department of Civil and Environmental Engineering, Politecnico di Milano, 20133 Milano, Italy; 2 Department of Environmental Science, Policy, and Management, University of California, Berkeley, CA 94720 USA; 3 Department of Civil and Environmental Engineering and Earth Sciences, University of Notre Dame, Notre Dame, IN 46556 USA; 4 Department of Ecosystem Science and Sustainability, Colorado State University, Fort Collins, CO 80523 USA; 5 Department of Soil and Crop Sciences, Colorado State University, Fort Collins, CO 80523 USA; 6 Department of Geography and Spatial Sciences, University of Delaware, Newark, DE 19716 USA; 7 Department of Plant and Soil Sciences, University of Delaware, Newark, DE 19716 USA; 8 Department of Environmental Policy Analysis, Institute for Environmental Studies (IVM), Vrije Univeristeit Amsterdam, Amsterdam, The Netherlands

**Keywords:** Environmental impact, Hydrology

## Abstract

The ongoing agrarian transition from smallholder farming to large-scale commercial agriculture promoted by transnational large-scale land acquisitions (LSLAs) often aims to increase crop yields through the expansion of irrigation. LSLAs are playing an increasingly prominent role in this transition. Yet it remains unknown whether foreign LSLAs by agribusinesses target areas based on specific hydrological conditions and whether these investments compete with the water needs of existing local users. Here we combine process-based crop and hydrological modelling, agricultural statistics, and georeferenced information on individual transnational LSLAs to evaluate emergence of water scarcity associated with LSLAs. While conditions of blue water scarcity already existed prior to land acquisitions, these deals substantially exacerbate blue water scarcity through both the adoption of water-intensive crops and the expansion of irrigated cultivation. These effects lead to new rival water uses in 105 of the 160 studied LSLAs (67% of the acquired land). Combined with our findings that investors target land with preferential access to surface and groundwater resources to support irrigation, this suggests that LSLAs often appropriate water resources to the detriment of local users.

## Abstract (web summary)

Water scarcity associated with large-scale land acquisitions is exacerbated by adoption of water-intensive crops and expansion of irrigation, which in turn increases rival water uses.

## Introduction

As a major determinant of agricultural production, water is a central target of agribusiness investments aimed at gaining access and control of this finite resource^1–4^. Unlike other natural resources such as timber or minerals, the physical transport of water is difficult and expensive, and its agricultural use therefore predominantly occurs locally^5,6^. Appropriation of water often takes place through crop production on land with suitable access to either rainwater for rainfed agriculture or surface water bodies and aquifers for crop irrigation^7^.

Because water rights are inseparable from the land in many regions, they remain inherently tied to land rights, as an appurtenance to land overlying an aquifer or abutting a surface water body. Water is consequently often acquired through land ownership or long-term land leases and concessions^3,7^. Research on large-scale land acquisitions (LSLAs) has long recognized that water is often a major determinant of land investments^1,8^. Thus, the key to understanding the ongoing surge in LSLAs for commercial farming is an accurate assessment of the underlying hydrological drivers and implications^7^.

Transnational large-scale land investments for agriculture have expanded rapidly in low- and middle-income countries, with agribusiness corporations and financial actors acquiring over 50 million hectares of arable land across more than 100 countries since 2005 in what is often described as a “global land rush”^9–12^. LSLAs have been promoted as a mechanism to support rural development through the increased input of financial capital, job creation, agricultural technology transfers, and gains in agricultural productivity, but these developments might come at the expense of reducing water access for local farmers and their future ability to irrigate.

Indeed, as LSLAs are generally implemented for agribusiness development, investors often seek to utilize local water resources to support irrigation and increased productivity within their acquired lands. Recent studies have examined the hydrological component of LSLAs^7^ and quantified the expected water use by intended crops^13^. This evidence that investors may utilize large volumes of water to support the production of export-bound cash crops within LSLAs is potentially problematic for local food security and livelihoods^14,15^, particularly if land investors selectively target areas based on hydrological conditions. This can cause the emergence of conflicts and social and political instability^16^. While previous studies have discussed and quantified the amount of blue water needed to meet irrigation water requirements in individual large-scale land deals^13,17^, a comprehensive analysis of the impact of these water appropriations on local water security and competition is still missing.

### Water scarcity and competition

**Fig. 1:**
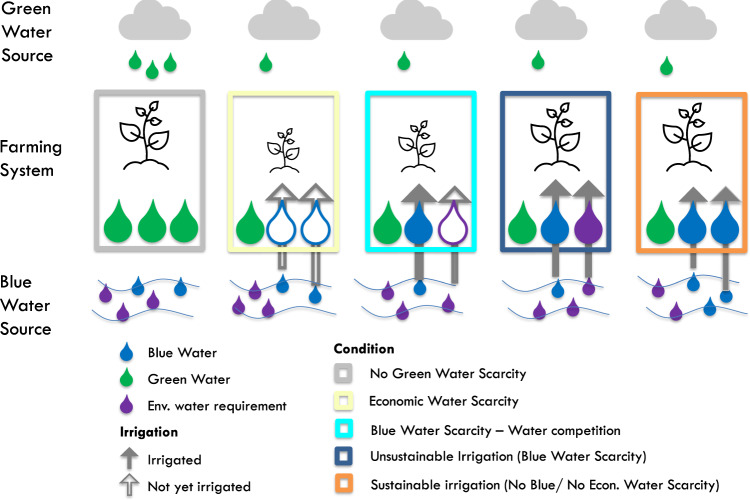
Water use and competition for different conditions of green (GWS), blue (BWS), and economic (EWS) water scarcity. Gray box: No GWS. Sufficient green water is available for rainfed farming. Yellow box: GWS with EWS and no BWS. Irrigation is needed and enough blue water is available for sustainable irrigation, but irrigation infrastructure is missing; crop-water needs can be met through the implementation of irrigation systems, channels, wells, and other irrigation infrastructure. Light and dark blue boxes: GWS with BWS; irrigation is needed but sufficient blue water is not available to satisfy irrigation needs. These conditions lead to either competition for water because of its limited availability (light blue box) or to meeting crop-water requirements at the expenses of environmental flows (“unsustainable irrigation”, dark blue box). Orange box: GWS but no BWS and no EWS (sustainable irrigation); in these conditions, crop yields can be enhanced by irrigation and both blue water and infrastructure are available to practice sustainable irrigation.

Agricultural water consumption includes both rainwater that infiltrates into the ground and is held within the root zone (green water), and irrigation water extracted from aquifers and surface water bodies to meet crop-water needs (blue water). While green water access is inherently tied to the transferred land, blue water withdrawals require ready access to aquifers and surface water bodies, as well as investments in infrastructure such as wells, canals, storage infrastructure, and conduits. Irrigation is needed only if green water is unable to meet the crop-water requirements, a condition referred to as “green water scarcity”^18,19^ (Fig. 1). In that case, irrigation is feasible only if blue water resources are both locally available and accessible. Water use for irrigation can be considered sustainable if irrigation water requirements can be met without compromising environmental flows or depleting groundwater resources. Conditions of “blue water scarcity” arise when blue water resources are insufficient to meet local irrigation needs (Fig. 1). These conditions are associated with a potential competition for water with the environment or other water users, which could lead to unsustainable or inequitable water appropriation^19–21^.

In fact, if investments occur in regions where blue water is scarce, water resources are insufficient for all users to (sustainably) irrigate their land. Water use then becomes “rival”, i.e., one farmer’s water consumption diminishes another local farmer’s ability to irrigate, and water competition ensues. Under these conditions, preferential access to blue water (in terms of physical proximity, upstream location, possession of water concessions, or availability of infrastructure for withdrawals) allows agribusiness investors to use blue water at the expense of local farmers or other users who have more limited access or lack the necessary economic resources and technical infrastructure.

These direct appropriations of blue water resources associated with LSLAs in blue water-scarce areas are very likely to represent “water grabs”. The normative interpretation of this diagnosis comes from the awareness that an imbalance of powers between local farmers and land investors has consistently led to a violation of basic ethical standards such as when local commons are appropriated through dynamics of dispossession by LSLAs^22^ or when water is appropriated in countries with high levels of malnourishment and physical water scarcity^4^.

The fact that water is physically available does not necessarily imply that it can be readily used to irrigate. This can occur under a situation of “agricultural economic water scarcity” in which blue water is available but a dearth of infrastructure prevents it from being extracted, stored, and conveyed at the time and location where it is needed^19^ (Fig. 1). It has been argued that land investments would allow for crop yield gap closure (i.e., the difference between current and attainable crop yields) in regions where irrigation infrastructure has historically been lacking^23,24^. It is therefore important to evaluate whether the acquired land was irrigated prior to the acquisition or whether irrigation infrastructure has been subsequently developed. While there is an emerging understanding of how LSLAs impact local crop production and food security^15^, the specific role of irrigation infrastructure still needs to be clarified.

Investments in irrigation infrastructure could mitigate agricultural economic water scarcity and contribute to closing the yield gap if blue water is abundant. However, if blue water is already scarce, new large-scale irrigation infrastructure would increase competition for water in local communities. We evaluate the propensity for LSLAs to fall in either of these two categories using a sample of 160 georeferenced foreign large-scale land investments (see Supplementary Table S1 and Supplementary Fig. S1) that account for 4.1 million hectares across 195 locations. We estimate the relative prevalence of green, blue, and economic water scarcity across the considered land deals. Because water scarcity is jointly determined by climate and agricultural practices, the water implications of LSLAs also depend on crop cover and crop type, and on how they change after the acquisition. A region can be adequate for rainfed agriculture by traditional smallholders, and successively become green water-scarce and blue water-scarce as agricultural developments associated with the LSLAs (i.e., changes in crop types and cropland expansion) are implemented. This temporal dimension is accounted for by considering three scenarios.

We first estimate preexisting water scarcity conditions to determine whether competitive water use (blue water scarcity) or irrigation investment deficit (economic water scarcity) was already prevalent before the land acquisitions (before acquisition scenario), all of which were concluded or put in production after 2000. We evaluate whether these conditions might have guided investors to target specific regions for acquisitions. Second, we leverage recent remote sensing estimates of the current cropland area (current use scenario) within the land deals to determine whether blue and economic water scarcity were amplified or attenuated after the implementation of each LSLA. Third, we estimate the hypothetical water needs associated with the expansion of the intended cultivated crops across the acquired land (100% cultivated scenario). In doing so, we disentangle the confounding effects of cropland expansion and crop type transition associated with LSLAs. Using these estimates to evaluate blue and economic water scarcity allows us to examine whether LSLAs aggravate water competition, or alleviate agricultural water scarcity. Finally, we use two specific deals in Ethiopia selected as representative examples to investigate in detail the hydrological dynamics associated with LSLAs and show how water scarcity is induced locally and how spillover effects play out in downstream areas.

### Pre-existing water availability conditions and targeting of land

We first used remote sensing estimates of crop cover, crop type information from agricultural censuses, and historical climate data to estimate crop-water requirements prior to LSLAs (before acquisition scenario) using an established crop-water model (see “Methods” section). Conditions of green, blue, and economic water scarcity were assessed by comparing the crop-water demands at each location to the local renewable water availability, which accounts for environmental flows, and the presence of irrigation equipment^19^.

**Fig. 2:**
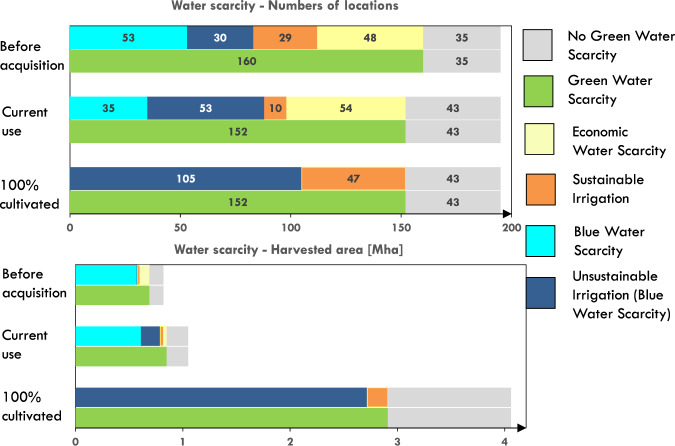
Green, blue, and economic water scarcity before large-scale land deals (before acquisition, i.e., with the areas and crops cultivated before the acquisition), in the current scenario (current use, i.e., current cultivated area and intended crops) and under potential cultivation expansion (100% cultivated, i.e., entire acquired area cultivated with intended crops). A location is classified as green water scarce if rainfall alone is not enough to meet 90% of the crop-water requirements^19^ It is found that thirty-five land deal locations (≈134,000 ha) were not affected by green water scarcity and were therefore suitable for rainfed production. An additional 83 locations (encompassing ≈ 578,000 ha) were both green water-scarce and blue water scarce (i.e., unable to sustainably meet the water requirement of pre-existing crops). Irrigation was found to be practiced (likely unsustainably) in 30 out of those 83 locations (≈12,000 ha). In the 77 remaining locations (≈114,000 ha) green water was scarce, but blue water resources were sufficient to meet the irrigation demand of pre-existing crops; only 29 of these locations, however, were equipped to some extent with irrigation systems (≈30,000 ha), leaving 48 locations (≈100,000 ha) in conditions of agricultural economic water scarcity (i.e., irrigation was not practiced despite the availability of blue water). Therefore, crop production in these areas would benefit from increased investments in irrigation infrastructure and would not compete for water resources with other farmers in the area.

Out of more than 4 million ha of contracted areas included in this study, only about 825,000 ha (i.e., ≈20%) were harvested (3.1% of which were irrigated) before the date of land acquisition (Fig. 2, before acquisition scenario). Results indicate that 16% of the area that was cultivated before the acquisition did not need irrigation (with the pre-existing crops) (i.e., no green water scarcity) while 14% of that land was irrigated and had sufficient blue water resources to meet the crop blue water requirements (i.e., that land was affected by green water scarcity but no blue water scarcity). In the remaining 70% of the area, even if the pre-existing crops needed irrigation, conditions of blue water scarcity did not allow all farmers to practice irrigation. These conditions may potentially lead to the emergence of competition for water (Fig. 1 and Supplementary Fig. S1).

The total annual amount of blue water used before acquisitions was 0.045 km^3^ (Supplementary File S3), mostly for the irrigation of rice (27% of total water use), sugarcane (8%), coffee (9%), maize (7%) and wheat (6%) (Supplementary Fig. S2). The additional amount of blue water needed to close the yield gap in cultivated green water-scarce areas would be 1.5 km^3^ y^−1^.

**Fig. 3:**
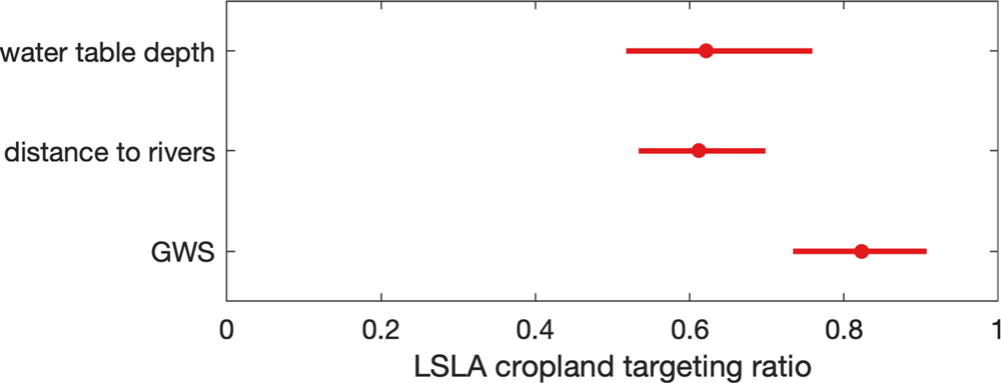
Preferential targeting of agricultural lands for LSLAs. Targeting ratios compare the water table depth, distance to rivers, and green water scarcity of the deal location to cropland areas within each targeted country. A ratio lower than one indicates that deals have lower-than-average values of a particular characteristic (e.g., shallower-than-average water table depth). Dots indicate the mean targeting ratio and bars are 95% bootstrapped confidence intervals.

To evaluate whether LSLAs have preferentially targeted locations with valuable water resources, we define a targeting ration that compares the water table depth, distance rivers, and green water scarcity at deal locations with average cropland characteristics within deal countries. We found that LSLAs target croplands located in areas with shallower-than-average water table depth and shorter-than-average distance to rivers (i.e., the targeting ratio is less than one, Fig. 3). However, deals also preferentially target areas with lower-than-average green water scarcity, meaning they occur on land that is more suitable for rainfed production than typical croplands within targeted countries (Fig. 3). Together, these results suggest that hydrological conditions, specifically proximity to freshwater resources for irrigation and soil water for rainfed production, may influence what areas are targeted by investors for LSLAs. Notably, in more than half of the deals (85 out of 160), the closest river flows directly through the acquired land (Supplementary Table S2).

### Existing scarcity and competition over water resources

To determine crop-water requirement under current conditions (in which some LSLAs have been put to productive use and others have not), we combined remote sensing estimates of current crop cover with crop types intended by investors^12^. About 20% of the harvested area (0.2 Mha) was identified as undergoing commercial farming through visual analysis of high-resolution imagery (see “Methods” section). For the corresponding land deals, we assumed that the intended crop types were distributed equally across the currently cropped areas and irrigated to satisfy crop-water demand. The remaining 80% of the cultivated land appears to remain used by smallholder farmers or unfarmed, where we assumed identical crop types and irrigation practices as in the previous (Before acquisition) scenario for each deal (see “Methods” section).

We find that in the period between 2000 and 2015 cultivated areas expanded to 26% of the acquired land (current use scenario). The expansion of cultivated areas and the shift to “intended crops” reshaped the distribution of areas affected by green, blue, and agricultural economic water scarcity. In particular, we found a 15-fold increase in irrigated areas affected by blue water scarcity (0.18 Mha, in 53 locations, Fig. 2, current use scenario), collectively consuming 0.5 km^3^ of irrigation water per year. If all currently cultivated land was irrigated (not only the commercial farms identified through visual analysis), the area affected by blue water scarcity would increase to 0.8Mha in 45% of the sampled locations (*n* = 88, Fig. 2, current use scenario). Such an increase in blue water scarcity, which is mostly found in Africa (Supplementary Fig. S2) could induce competition for irrigation water with nearby farmers.

From a hydrological point of view, our results show different impacts of LSLAs on water resources. While in some areas capital investments and irrigation infrastructure development associated with LSLAs lead to a general reduction of agricultural economic water scarcity (Fig. 1 moving from the yellow to the orange box), in 23 cases (≈168,000 ha) land acquisitions are exacerbating conditions of blue water scarcity (Fig. 1, blue boxes). Specifically, in 64 cases the presence of LSLAs might have resulted in increased crop production (due to irrigation expansion) without compromising local water availability for adjacent or downstream users (Fig. 2, current use scenario, yellow and orange). Visual analysis (see “Methods” section) allowed us to confirm the development of pivot irrigation in 10 of these cases. Conversely, in 88 blue water-scarce locations, irrigation introduced by land investors might have caused (current) competition over water resources and associated blue water appropriation (Fig. 2, current use scenario, blue and light blue). Our visual analysis confirms the presence of pivot irrigation in 53 of these locations (Fig. 2, current use scenario, blue).

### Prospective aggravation of water scarcity and competition

Lastly, we considered the scenario in which agribusiness investors expand the cultivation of intended crops to the entire acquired area (100% cultivated scenario) to maximize production, using irrigation where needed. Under this scenario, we assumed that irrigation water needs are completely met across the entire acquired land and that conditions of agricultural economic water scarcity are not present. We estimated that 8.1 km^3^ y^−1^ are necessary for irrigation to close the crop yield gap, a 180 fold increase relative to current conditions (Supplementary Figs. S1, S2 and Supplementary File S3). Confirming previous studies, we find that irrigation water needs are particularly high (5.6 km^3^ y^−1^) in Africa^7,13^. The 100% cultivation scenario shows a substantial increase in blue water scarcity (2.7 Mha, across 67% of the acquired area in 105 locations, Fig. 2, 100% cultivated scenario, blue), compared to the “before deal” and “current” scenarios. In these locations, which account for 54% of the sampled land deals, the irrigation water requirements associated with the intended agribusiness expansion cannot be sustainably met, which entails competition for blue water with local users and the environmental flow requirements of the area. In contrast, sufficient blue water is available to sustainably support the intended agribusiness expansion in only 47 locations, which represent a mere 5% of the land area in the considered sample (Fig. 2., 100% cultivated scenario, orange). The potential blue water appropriation associated with LSLAs, here estimated at 8.1 km^3^ y^−1^, is by far smaller than the volumes needed to support global agriculture (i.e., Jaramillo et al.^25^ and Rost et al.^26^). However, these water appropriations may have strong local impacts on rural communities and the environment. Moreover, water withdrawals were estimated under two different irrigation scheme scenarios. By adopting an irrigation system with the same efficiencies as those currently existing (on average) in the same country^27^, we estimate 27.7 km^3^ y^−1^ of blue water withdrawals under 100% cultivation in LSLA areas. We estimate 10.9 km^3^ y^−1^ of blue water withdrawals assuming the average efficiency of a sprinkler irrigation system (i.e., 75%^28^) (Supplementary Table S4).

The expected increase in green and blue water scarcity compared to pre-acquisition conditions (100% cultivated vs Before acquisition scenarios in Fig. 2) results both from the expansion of the harvested areas and the shift to the intended crops. To assess to what extent the expected increase in blue water scarcity was induced by the shift in crop types, we considered an additional scenario in which the entire acquired land is cultivated with the crops planted before the acquisition (i.e., the 100% cultivated area scenario is planted with crops from before acquisition scenario). We found that the blue water needed to meet the water requirements of the before acquisition crops is 5.6 km^3^ y^−1^, which is about 30% less than in the 100% cultivated scenario (Supplementary Fig. S3). This result indicates that LSLAs induce blue water scarcity not only through an expansion of the cultivated land but also through shifts to more water-demanding crops. Specifically, cash crops with relatively high water requirements, such as oil palm and sugarcane, account for 40% of the total additional blue-water needs, followed by maize (12%), soybean (9%), and wheat (9%) (Supplementary Fig. S2).

## Discussion

There are concerns that the increasing transnational control of water resources through the acquisition of land by agri-food transnational land investments might collide with the current needs and future agricultural development opportunities of local farmers. Growing evidence shows that LSLAs often negatively impact the environment^29,30^, threaten and displace best agricultural practice^31^, rural livelihoods^32,33^ and local food security^14,15^ and rely on a process of agrarian change that is often counter to achieving multiple Sustainable Development Goals^34^.

This study adds to this literature from the perspective of water resources. We find little evidence that LSLAs can improve crop yields through irrigation sustainably across the majority of land deals. In contrast, we show that much of the agricultural expansion associated with LSLAs will take place in water-scarce regions. The associated increase in water use for irrigation is fueled by both cropland expansion and the transition towards water-intensive crop types and will occur in competition with local water needs. These findings—along with the fact that many LSLAs are being implemented along river banks or at a relatively short distance from water courses and groundwater (Supplementary Table S2)—indicates that transnational land investments are facilitating water appropriation and grabbing^3,4,22,35^. These effects on water availability for withdrawal add onto other issues known to arise even if sufficient water is available (including effects of LSLA’s on water quality, water access, and freshwater fisheries) (i.e., Williams et al.^36^, Fonjong et al.^37^, Friis et al.^38^, Johansson et al.^39^, Adams et al.^40^) and likely underestimate water implications of LSLA’s on local communities. More than 2 million people live within the boundary of LSLAs. Thus, the expansion of transnational large-scale land acquisitions into water-scarce areas raises environmental and ethical concerns along multiple dimensions that are supported by our findings. First, investors preferentially acquire locations that are hydrologically preferable over local average cropland conditions. Removing this land from local use increases the average water scarcity faced by local farmers. Second, the establishment of commercial agriculture in these areas is expected to accelerate the development of large-scale irrigation, leading to blue water appropriations that, in the majority of the cases included in this study, will exacerbate rivalry and competition with local farmers, thereby enhancing potential threats and impacts on local systems of production, food security, rural livelihoods, and ecosystems (e.g., Dell’Angelo et al.^4^, Dell’Angelo, D’Odorico, & Rulli^22^; Müller et al.^15^). Notice that, while irrigation may affect local precipitation patterns if it occurs in sufficiently large areas^41^ (e.g., Segal et al.^42^, Puma and Cook^43^, Alter et al.^44^, DeAngelis et al.^45^, Lo and Famiglietti^46^), these effects are not accounted for in this study. Third, intended crop types imply transitions away from locally adapted staple crops and towards water-intensive commercial crops. These transitions account for a substantial portion of the estimated increased water use within land deals. Because the intended crops are mainly destined for the export market^15^, the associated virtual water will likely bypass local communities and fail to provide positive livelihood and food security benefits. Finally, water use within the acquired land may not only affect water availability within the investment area or adjacent farms but also compete with the water demands of downstream users through upstream blue water withdrawals for irrigation as well as a reduction in downstream runoff resulting from increased green water uses at upstream locations (Box 1). This water competition will compound and further propagate if downstream smallholder farmers adapt to these new water scarcity conditions by themselves increasing their reliance on potentially unsustainable irrigation.

**Fig. 4:**
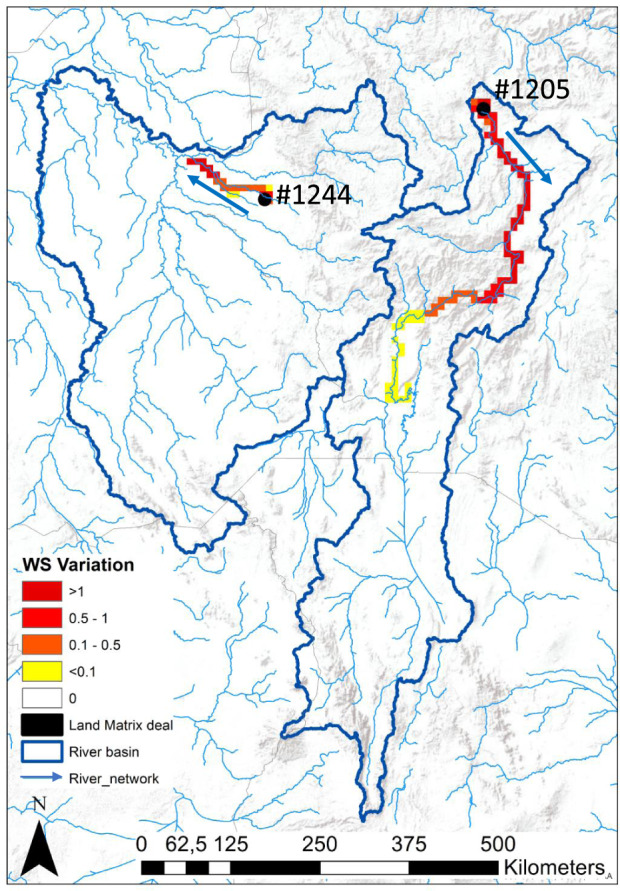
Water scarcity variation for the two deals (#1205 and #1244) located in the Oromya region in the Omo River basin and in the Gambella region in a tributary of the Nile river during the month of December. Both deals have a size of about 15,000 ha, with maize, sugarcane, cotton and sunflower as the intended crops.

Box 1 Downstream implications of water appropriation by LSLAs To illustrate the emerging competition for water induced by LSLAs we considered the specific example of how upstream agribusiness development reduces water availability at downstream locations. We examined more closely the case of two land deals irrigated with surface water in North West Ethiopia (Box 1)^12^. During the dry season, the irrigation of the selected large-scale land deals enhanced water scarcity in the downstream areas, with the strongest impacts occurring in the months of December and January (Fig. 4 and Supplementary Fig. S4). Water scarcity increases by more than 10% in about 23,000 ha and 2300 ha of cultivated land downstream of the two land deals (1205 and 1244, respectively) during the worse month. Because the areas downstream of these two deals are mainly under rainfed production, the impact of upstream water appropriations by LSLAs is not perceived by local farmers today but limits their future agricultural development opportunities (i.e., future blue water grabbing), while also affecting pastoralist communities in the area. These two cases represent an illustrative example of how the appropriation of blue water resources by large-scale land investors could prevent future irrigation of downstream areas by local farmers.

## Concluding remarks

Reconciling increasing water demands in a world of finite water resources represents a fundamental challenge for sustainable development. Agricultural production represents the most water-intensive sector, accounting for about 90% of the global water consumption^47^. In response to worsening hydro-climatic crises and advancing severe water scarcity^19,48^ agribusiness investors are becoming increasingly interested in controlling water resources for agriculture, particularly through large-scale land acquisitions.

The introduction of new irrigation infrastructure, especially in the Global South should be carefully assessed. Robust water accounting, caps on water extractions, measurements, valuation of socio-hydrological trade-offs, assessment of “winners” and “losers” and of uncertainties are all important measures that should be discussed for an integrated governance of scarce water resources in the context of agricultural development^49^.

Overall, our findings indicate that LSLAs contribute substantially to the implementation of irrigation infrastructure and the subsequent increasing demand for blue water resources, with potentially profound social and hydrological consequences for local users. To date, recent advances in the understanding of the water dimension of LSLAs are still not matched by a direct and strong policy attention. So far only some shy attempts have been made to regulate, mainly on a voluntary basis, land investment in the Global South^50,51^, while the governance of transnational water appropriations and water grabbing has been overlooked by formal international policy deliberations, pointing to the need for policies accounting for hydrological constraints, rural livelihoods, and principles of water justice.

## Methods

### Land deals

The set of deals used in this study were provided by the Land Matrix database^12^, which contains information about international (and domestic) land acquisitions and, to our knowledge, represents the most extensive data set for LSLAs globally. The entire database contains nearly 2000 land deals with a variety of intended uses falling outside the scope of this study. We, therefore, filtered the deals to those that are (1) marked as “contracted,” “in startup phase,” or “in production” after 2000, (2) are intended for agriculture and cover more than 200 ha, (3) involved a transfer of rights (via sale, lease, or concession) to foreign commercial users, and (4) contain reliable coordinates for the deal location. The final sample contained 160 deals, 37 of which contained multiple locations (i.e., 197 locations in total) across 4 macro-regions and 39 countries (Supplementary Table S1). Each deal was marked with one or more intended uses. Most of the deals (58%) were intended for “food,” and smaller portions were intended for “nonfood” or “unspecified” agriculture (38%), “biofuels” (17%), “timber plantations” (11%), and “livestock” (12%). The spatial information provided by Land Matrix contains only the deal location and size, but not the boundaries of the deal. We, therefore, approximated deals as discs centered on the location coordinates provided. Most of them are located in Africa (73 deals accounting for 2.4 Mha), followed by Asia (43 deals, 0.58 Mha), Europe (33 deals, 0.53 Mha), and South America (11 deals, 0.50 Mha). The average deal size is about 26,000 ha. For each location, we approximated the spatial extent of the acquired land with a disc centered on the centroid and with the same area as reported by the Land Matrix. Information on the year when land deals were signed was available for about 80% of the LSLAs included in this study. Thirty-two land deals were signed or concluded in 2010 (20%), with 50% of the deals signed between 2008 and 2012. After 2012, a decreasing trend was observed and only 4 deals were signed in 2018. Additional details for the deals can be found in Müller et al.^15^. All of the deals and associated characteristics can be downloaded at 10.7274/r0-ycpf-qh53.

### Crop coverage and current irrigation

Crop coverage in the before deal (Before acquisition scenario) and current irrigation scenarios were calculated from two separate datasets. For the before acquisition scenario, we used the Global Land Cover Dataset (i.e., GlobCover), which provides a global land use map for 2005 at 300 m resolution. For the current irrigated scenario, we used the NASA Global Food Security-support Analysis data set (GFSAD30)^52^, which provides a global map of cropped and non-cropped pixels at 30 m resolution. The GlobCover data set provides four binned categories with respect to crop coverage (<20%, 20–50%, 50–70%, and >70%). To correct for biases in this data set, we aggregated the GFSAD data set to 300 m, binned the data into the same categories, and generated proportionality constants by comparing the aggregated (300 m and binned) data with the original 30 m data. These constants were then used to translate the GlobCover 2005 data into cropped areas within each deal. This approach was developed in Muller et al.^15^, and we refer to that manuscript for complete details. The deal locations, total areas, and cropped areas are provided in CSV format on https://curate.nd.edu/show/rv042r40b62.

For assessing current irrigation in the current use scenario, presence or absence of irrigation pivots was assessed visually using Google Earth Pro time series imagery for each individual deal. For the most recent image available for an individual LSLA, we recorded the year of the image (typically 2019 or 2020) and the presence or absence of irrigation pivots. We repeated this for the available image with year closest to year 2000. By comparing the two time steps, this method allowed for the broad identification of changes in the presence/absence of irrigation infrastructure within deal areas over the study period.

### Green and blue water demand

Green and blue water consumption were evaluated for harvested and intended crops for three different scenarios: before land acquisition, considering the current cultivated land and current irrigation, and considering a 100% cultivation expansion scenario in which the entire acquired land is cultivated with the intended crops as reported in Land Matrix and irrigation water needs are met. A crop-water model—WATNEEDS—was used to evaluate the occurrence of green and blue water scarcity conditions and to quantify the amount of irrigation water needed to fully meet the crop-water requirements under non-water-stressed conditions^53^. The model uses a soil water balance coupled with evapotranspiration estimates based on the Penman–Monteith method and accounting for the effects of water limitation and crop phenology through a water stress and crop coefficient as in Allen^28^. Specifically, spatially distributed, crop-specific maps of rainfed and irrigated areas were used^54^ to estimate crop-water use before land investments (before acquisition scenario, ca 2000). To do so, crop-specific cover estimates for each land deal were scaled to match the remotely sensed crop cover estimates prior to the deals reported in Muller et al.^15^. The crop-water model was also run considering the intended crops reported by the Land Matrix for each deal (current use and 100% cultivated scenario). When more than one intended crop was reported by the Land Matrix, an equal fraction of the cultivated area was assigned to each crop. In this analysis, we used the average climate parameters for 2011–2015, while crop coefficients, planting, and harvesting dates were based on Siebert et al.^55^. In the case of crops for which these data were not reported by Siebert et al.^55^, the crop parameters were taken from Chapagain et al.^56^. For intended crops reported by Land Matrix two different scenarios of irrigation were considered: a current scenario in which only a fraction of the acquired land is currently cultivated and part of it is irrigated accordingly to a visual inspection and the 100% cultivation expansion scenario, which entails the expansion of agriculture across the acquired land and the use of irrigation if the intended crops are affected by green water scarcity.

### Water scarcity assessment

Following Rosa et al.^19^, we assessed green water scarcity, blue water scarcity and agricultural economic water scarcity. Green water scarcity was expressed as the ratio between irrigation blue water requirements (or green water deficits) and crop-water requirements. Crops face green water scarcity when rainfed conditions cannot meet the 90% of the crop-water requirements^19^.

Monthly blue water scarcity was assessed by comparing water consumption for agricultural, domestic, and industrial needs in each reported location against the available blue water. Agricultural blue water consumption was assessed using WATNEEDS for the three considered scenarios: before the land acquisition (before acquisition scenario), intended crops with current irrigation conditions (current use scenario), and intended crops with ideal irrigation that allows for complete water gap closure. Domestic and industrial water consumption (5 × 5 arc minute resolution) were taken from Hoekstra and Mekonnen^47^ and considered homogeneous during the year. These volumes were added to agricultural consumption and kept constant both prior to the LSLAs (ca 2005, Before acquisition scenario) and after (ca 2015, current use and 100% cultivated scenarios). Monthly available water (5 × 5 arc minute resolution) for the period 2011–2015 period was calculated as the difference between monthly blue water flows generated in each grid cell or received from the upstream area taken from Sutanudjaja et al.^57^ and the environmental flow requirement. Upstream water consumption and its effect on downstream blue water availability was accounted for by considering all water uses (agriculture, domestic, and industry) for every grid cell. We also accounted for environmental flow, here defined as the minimum freshwater flow required to sustain ecosystem functions and estimated as a fraction (80%) of “natural” (i.e., with no anthropogenic disturbance) monthly blue water flow^58^. Thus, environmental flows are not available for human water consumption. This methodology to assess blue water scarcity has been extensively used and validated in studies aiming at analyzing the influence of agricultural production on water resources^19,59–61^. Losses associated with low irrigation efficiencies (i.e., with the difference between water withdrawal and consumption) are not accounted for because that water is not evapotranspired but turns into surface and groundwater runoff and is therefore available for downstream uses, except for the case of coastal areas. Agricultural economic water scarcity has been calculated over croplands not equipped for irrigation, facing water scarcity but no blue water scarcity. Irrigation infrastructure has been computed by considering the irrigated areas (see above) for the scenario before land acquisition and the commercial farming for the current scenario.

### Cropland targeting of water resources

Following Muller et al.^15^, we assessed the preferential targeting of land using targeting ratios that compare specific water resource attributes of deal locations (distance to rivers, water table depth, and green water scarcity) compared to the average attribute value across all cropland of the corresponding country. Green water scarcity from Rosa et al.^19^ is measured in months of scarcity during growing seasons, varying from zero to a maximum of three. We used the river network map by Grill et al.^62^ to evaluate the distance between the deal’s centroid and the closest river. Only rivers with an upstream catchment basin of at least 1000 km^3^ were considered. This cutoff allowed us to focus on perennial rivers. We also evaluated the sensitivity of our results to this threshold value and found that a decrease to 500 km^2^ or an increase to 2000 km^2^ led to negligible changes (i.e., <1%). Similarly, the average groundwater depth was extracted for each land deal from the groundwater table map by Fan et al.^63^.

Out of the 160 study deals, only 24 are reported to be irrigated (16 with surface irrigation). The majority are located in Africa (12), with only 3 and 1 in Eastern Europe and Asia, respectively. Thus, we focus on two deals located in the central west part of Ethiopia (i.e., deal 1205 and 1244 in the Land Matrix). The two deals are located in the Oromya region in the Omo River basin and in the Gambella region in a tributary of the Nile river (Box 1), a region particularly affected by land acquisition with large irrigation projects^64^ and characterized by at least 3 months of blue water scarcity. Both deals have a size of about 15,000 ha, with maize, sugarcane, cotton, and sunflower as the intended crops. Flow propagation within the basin was assessed using the crop-water model and flow directions from the HydroSheds data set^65^. By carrying out this analysis, we do not only account for the local consumption of each deal, but we propagate the effect of such water use, with the potential to affect downstream activities. These appropriations of water can exclude other farmers from the use of that blue water.

### Uncertainty and limitations

Our estimates are subject to a range of uncertainties associated with the assumptions that we make and the data that we use. First, some deals might be merely speculative (and may not be actively put under production), despite being reported as “in production” in the Land Matrix data set. Other deals might be used for purposes other than what was intended^12^. Second, we computed green and blue water consumption to satisfy the crop-water requirements of intended crops as reported by Land Matrix data set, but the actual extent of irrigation within each deal is uncertain. While Land Matrix currently reports that irrigation is intended in 24 out of the 160 studied deals, but this might underestimate the true number of irrigated land deals. In our current use and 100% cultivated scenarios, we assumed that all currently cropped and acquired areas are fully irrigated, respectively. The possible overestimation of crop-water use that this implies might be mitigated by the fact that the amount of blue water considered in this study corresponds to the consumptive use of blue water. This represents only the amount of water needed by the plants and excludes losses associated with the efficiency of the irrigation system used (i.e., transportation, evaporation, incorrect water application). Third, different types of crops than the one reported in Land Matrix might have been harvested, thus affecting the demand for blue water or, again, only a fraction of the acquired area might have been used for crop production. We also set blue water requirement for trees, (including eucalyptus and rubber) to 0, under the assumption that tree plantations are seldom irrigated. Therefore, our analysis provides a more conservative assessment of water competition resulting from LSLAs. Fourth, we acknowledge that surface water storage (through dams and other infrastructures) can help to store water for drier months, thus affecting the temporal distribution of water and the severity of blue water scarcity. Small reservoirs, as well as big dams, are currently being built all around the world, and their effect on blue water scarcity is neglected in our analysis. Lastly, using groundwater as a source for blue water could lead to a wide range of future problems that are not discussed within the context of this analysis. Issues associated with groundwater irrigation use are difficult to detect until the water table has been depleted, unless a dense network of observation wells are deployed. The consequences of groundwater depletion are likely to accrue before the problem is detected. Given the potential for LSLAs to consume groundwater and increase blue water scarcity, a potential solution for future land deals could be to require regular groundwater monitoring within the deal.

## Supplementary information

Supplementary Information
