# Supplementary material for: Competition for water induced by transnational land acquisitions for agriculture
Source: Nat Commun. 2022 Jan 26;13:505. doi: 10.1038/s41467-022-28077-2 (PMC8791946; doi:10.1038/s41467-022-28077-2)
Supplement: Supplementary file 1 — Supplementary Information [file 41467_2022_28077_MOESM1_ESM.pdf]

## Supplementary tables and figures

**List:**

Table S1 Deals included
Table S2 River distance and mean groundwater table depth
Table S3 Blue water needed in each deal [m3] under the three scenarios
Table S4 Blue water withdrawal in the 100% cultivated scenario
Figure S1 Large scale land acquisitions distribution in the before acquisition (top) and 100% cultivation (bottom) scenarios
Figure S2 Blue water (BW) volumes by crops under the three scenarios
Figure S3 Water scarcity under the 100% cultivated scenario and in a hypothetical scenario in which the entire acquired land is cultivated with the crops harvested before LSLAs.
Figure S4 Water scarcity variation for the two deals (#1205 and #1244) located in the Oromya region

**Supplementary table S1.** Deals included (Data are taken from Land Matrix)

| Land Matrix ID | Target country | Target region | Intended size [ha] | Contracted size [ha] | In prod. size [ha] | Intention of investment | Crops |
|---|---|---|---|---|---|---|---|
| **23** | **Asia** | **Cambodia** | 5000 | 5000 | 0 | ###Biofuels | ###Cassava (Maniok) |
| **33** | **Asia** | **Cambodia** | 769 | 769 | 0 | ###Non-food agricultural commodities | ###Rubber tree |
| **35** | **Asia** | **Cambodia** | 7200 | 7200 | 0 | ###Non-food agricultural commodities, Timber plantation (for wood and fibre) | ###Accacia, Rubber tree |
| **42** | **Asia** | **Cambodia** | 4821 | 4821 | 1001 | ###Food crops, Livestock, Timber plantation (for wood and fibre) | ###Cashew, Teak |
| **105** | **Asia** | **Cambodia** | 8100 | 8100 | 7000 | ###Non-food agricultural commodities | ###Rubber tree |
| **138** | **Asia** | **China** | 200 | 200 | 0 | ###Livestock, Industry | 0 |
| **427** | **Asia** | **Philippines** | 350 | 350 | 200 | ###Food crops | ###Corn (Maize) |
| **614** | **Asia** | **India** | 3642 | 3642 | 0 | ###Biofuels, Renewable Energy | ###Bamboo, Trees (unspecified) |
| **746** | **South America** | **Brazil** | 300000 | 300000 | 300000 | ###Food crops | ###Cotton, Corn (Maize), Soya Beans, Wheat |
| **767** | **South America** | **Brazil** | 100000 | 100000 | 100000 | ###Timber plantation (for wood and fibre) | ###Eucalyptus |
| **877** | **South America** | **Paraguay** | 2859 | 2859 | 760 | ###Food crops, Conservation | ###Corn (Maize), Eucalyptus, Soya Beans, Sugar Cane |
| **1037** | **Europe** | **Ukraine** | 242400 | 242400 | 0 | ###Biofuels, Food crops, Livestock | ###Barley, Corn (Maize), Soya Beans, Sugar beet, Sun Flower, Wheat |
| **1064** | **South America** | **Argentina** | 37273 | 37273 | 8000 | ###Food crops, Livestock | ###Rice, Soya Beans |
| **1065** | **South America** | **Argentina** | 17495 | 17495 | 17495 | ###Food crops, Livestock | ###Corn (Maize), Soya Beans, Wheat |
| **1066** | **South America** | **Argentina** | 10848 | 10848 | 10848 | ###Food crops | ###Corn (Maize), Soya Beans, Wheat |
| **1067** | **South America** | **Argentina** | 15451 | 15451 | 15451 | ###Food crops | ###Corn (Maize), Soya Beans, Wheat |
| **1113** | **Africa** | **Benin** | 5200 | 5200 | 0 | ###Biofuels, Food crops, Renewable Energy | ###Sugar Cane |
| **1141** | **Africa** | **Cameroon** | 11980 | 11980 | 0 | ###Biofuels, Food crops, Renewable Energy | ###Sugar Cane |
| **1166** | **Africa** | **Congo, Rep.** | 470000 | 470000 | 570 | ###Biofuels, Agriculture unspecified, Timber plantation (for wood and fibre), Renewable Energy | ###Oil Palm |
| **1173** | **Africa** | **Egypt, Arab Rep.** | 20000 | 20000 | 0 | ###Food crops | ###Dill, Alfalfa, Corn (Maize), Potatoes, Wheat |
| **1205** | **Africa** | **Ethiopia** | 15000 | 15000 | 1200 | ###Biofuels, Food crops, Agriculture unspecified, Renewable Energy | ###Corn (Maize), Oil Palm, Rice, Sugar Cane |
| **1218** | **Africa** | **Ethiopia** | 10000 | 10000 | 0 | ###Food crops | ###Cereals (unspecified), Pulses (unspecified), Rice |
| **1244** | **Africa** | **Ethiopia** | 14000 | 14000 | 1800 | ###Biofuels, Food crops, Renewable Energy | ###Corn (Maize), Cotton, Rice, Sugar Cane, Sun Flower, Teff |
| **1322** | **Africa** | **Ghana** | 6750 | 6750 | 1400 | ###Biofuels | ###Jatropha |
| **1323** | **Africa** | **Ghana** | 100000 | 100000 | 1000 | ###Biofuels | ###Jatropha |
| **1388** | **Africa** | **Liberia** | 220000 | 220000 | 3000 | ###Non-food agricultural commodities, Agriculture unspecified | ###Oil Palm, Rubber tree |
| **1467** | **Africa** | **Mali** | 20000 | 20000 | 12000 | ###Biofuels, Food crops, Renewable Energy | ###Sugar Cane |

| Land Matrix ID | Target country | Target region | Intended size [ha] | Contracted size [ha] | In prod. size [ha] | Intention of investment | Crops |
|---|---|---|---|---|---|---|---|
| **1562** | **Africa** | **Mozambique** | 1800 | 1800 | 0 | ###Food crops | ###Rice (hybrid) |
| **1575** | **Africa** | **Mozambique** | 7500 | 7500 | 0 | ###Biofuels | ###Jatropha |
| **1652** | **Africa** | **Namibia** | 220 | 220 | 200 | ###Food crops | ###Grapes, Palms |
| **1684** | **Africa** | **Nigeria** | 10000 | 10000 | 4400 | ###Food crops, Industry | ###Cashew, Rice |
| **1721** | **Africa** | **Sudan** | 20492 | 20492 | 3800 | ###Food crops | ###Barley, Alfalfa, Corn (Maize), Wheat |
| **1777** | **Africa** | **Senegal** | 418 | 418 | 418 | ###Food crops | ###Tomatoes, Corn (Maize), Fruit (unspecified) |
| **1795** | **Africa** | **Senegal** | 1430 | 1430 | 900 | ###Food crops | ###Bean, Cereals (unspecified), Corn (Maize), Onion, Peanut (groundnut), Potatoes, Sweet Potatoes, Vegetables (unspecified), Pepper |
| **1798** | **Africa** | **Sierra Leone** | 23500 | 23500 | 12000 | ###Biofuels, Food crops, Renewable Energy | ###Cassava (Maniok), Sugar Cane |
| **1817** | **Africa** | **Sierra Leone** | 800 | 800 | 0 | ###Agriculture unspecified | ###Oil Palm |
| **1839** | **Africa** | **Tanzania** | 5818 | 5818 | 4178 | ###Food crops | ###Rice |
| **1976** | **Africa** | **Uganda** | 7591 | 7591 | 6225 | ###Agriculture unspecified | ###Oil Palm |
| **2024** | **Africa** | **Zambia** | 27087 | 27087 | 0 | ###Food crops, Livestock, Non-food agricultural commodities | ###Corn (Maize), Soya Beans, Wheat |
| **2053** | **Africa** | **Zambia** | 4094 | 4094 | 4094 | ###Food crops | ###Corn (Maize), Soya Beans, Wheat |
| **2241** | **Africa** | **Ghana** | 13058 | 13058 | 10000 | ###Food crops, Timber plantation (for wood and fibre) | ###Corn (Maize), Rice, Soya Beans, Teak |
| **2371** | **Africa** | **Sierra Leone** | 17812 | 17812 | 11057 | ###Non-food agricultural commodities, Agriculture unspecified | ###Oil Palm, Rubber tree |
| **2385** | **Africa** | **Mozambique** | 1000 | 1000 | 0 | ###Food crops | ###Tomatoes, Corn (Maize), Potatoes |
| **3008** | **Africa** | **Sierra Leone** | 1845 | 1845 | 1845 | ###Biofuels, Food crops, Renewable Energy | ###Cassava (Maniok), Sugar Cane |
| **3031** | **Africa** | **Mozambique** | 8789 | 8789 | 0 | ###Biofuels | ###Jatropha |
| **3272** | **Africa** | **Cote d'Ivoire** | 5000 | 5000 | 1900 | ###Agriculture unspecified | ###Oil Palm |
| **3389** | **Africa** | **Ghana** | 3500 | 3500 | 3500 | ###Timber plantation (for wood and fibre), For carbon sequestration/REDD | ###Teak, Trees (unspecified) |
| **3393** | **Africa** | **Ghana** | 400000 | 400000 | 60030 | ###Biofuels, Food crops, Non-food agricultural commodities, Renewable Energy | ###Tea, Coffee Plant, Food crops (unspecified), Oil Palm, Rubber tree, Sugar Cane |
| **3398** | **Africa** | **Ghana** | 50000 | 50000 | 0 | ###Biofuels | ###Jatropha, Oil Seeds (unspecified) |
| **3399** | **Africa** | **Ghana** | 10497 | 10497 | 40 | ###Food crops | ###Corn (Maize) |
| **3403** | **Africa** | **Congo, Rep.** | 68000 | 68000 | 0 | ###Timber plantation (for wood and fibre) | ###Eucalyptus |
| **3404** | **Africa** | **Ghana** | 10000 | 10000 | 250 | ###Timber plantation (for wood and fibre) | ###Eucalyptus, Pine, Teak |
| **3421** | **Asia** | **Cambodia** | 9014 | 9014 | 0 | ###Non-food agricultural commodities | ###Rubber tree |
| **3422** | **Asia** | **Cambodia** | 9656 | 9656 | 0 | ###Non-food agricultural commodities | ###Rubber tree |
| **3459** | **Asia** | **Cambodia** | 9114 | 9114 | 0 | ###Non-food agricultural commodities | ###Rubber tree |
| **3462** | **Asia** | **Cambodia** | 9773 | 9773 | 0 | ###Non-food agricultural commodities | ###Rubber tree |
| **3463** | **Africa** | **Liberia** | 8800 | 8800 | 0 | ###Agriculture unspecified, For carbon sequestration/REDD | ###Oil Palm |
| **3470** | **Asia** | **Cambodia** | 7900 | 7900 | 0 | ###Non-food agricultural commodities | ###Rubber tree |
| **3472** | **Asia** | **Cambodia** | 7591 | 7591 | 0 | ###Non-food agricultural commodities | ###Rubber tree |
| **3479** | **Asia** | **Cambodia** | 6695 | 6695 | 1404 | ###Non-food agricultural commodities | ###Rubber tree |
| **3484** | **Asia** | **Cambodia** | 5080 | 5080 | 30 | ###Non-food agricultural commodities | ###Rubber tree |
| **3486** | **Asia** | **Cambodia** | 1200 | 1200 | 0 | ###Biofuels, Food crops | ###Sugar Cane |
| **3503** | **Asia** | **Cambodia** | 9472 | 9472 | 0 | ###Non-food agricultural commodities | ###Rubber tree |
| **3510** | **Asia** | **Cambodia** | 6592 | 6592 | 0 | ###Non-food agricultural commodities | ###Rubber tree |
| **3525** | **Asia** | **Cambodia** | 9119 | 9119 | 1000 | ###Non-food agricultural commodities | ###Sugar (unspecified) |
| **3527** | **Asia** | **Cambodia** | 8400 | 8400 | 0 | ###Non-food agricultural commodities | ###Rubber tree |
| **3533** | **Asia** | **Cambodia** | 9015 | 9015 | 0 | ###Non-food agricultural commodities, Agriculture unspecified | ###Accacia, Rubber tree, Sugar Cane |
| **3534** | **Asia** | **Cambodia** | 8841 | 8841 | 500 | ###Non-food agricultural commodities, Agriculture unspecified, Industry | ###Rubber tree |
| **3539** | **Asia** | **Cambodia** | 10601 | 10601 | 0 | ###Non-food agricultural commodities | ###Rubber tree |

| Land Matrix ID | Target country | Target region | Intended size [ha] | Contracted size [ha] | In prod. size [ha] | Intention of investment | Crops |
|---|---|---|---|---|---|---|---|
| **3554** | **Asia** | **Cambodia** | 34007 | 34007 | 0 | ###Timber plantation (for wood and fibre), For carbon sequestration/REDD | ###Trees (unspecified) |
| **3593** | **Asia** | **China** | 145000 | 145000 | 0 | ###Food crops, Livestock, Industry | ###Corn (Maize), Rice, Soya Beans |
| **3612** | **Asia** | **Indonesia** | 2586 | 2586 | 2427 | ###Agriculture unspecified | ###Oil Palm |
| **3614** | **Asia** | **Indonesia** | 21500 | 21500 | 9424 | ###Agriculture unspecified | ###Oil Palm |
| **3618** | **Asia** | **Indonesia** | 35759 | 35759 | 0 | ###Agriculture unspecified | ###Oil Palm |
| **3640** | **Asia** | **Indonesia** | 12628 | 12628 | 0 | ###Agriculture unspecified | ###Oil Palm |
| **3641** | **Asia** | **Indonesia** | 7309 | 7309 | 0 | ###Agriculture unspecified | ###Oil Palm |
| **3715** | **Asia** | **Indonesia** | 7568 | 7568 | 736 | ###Agriculture unspecified | ###Oil Palm |
| **3761** | **Africa** | **Ghana** | 761 | 761 | 0 | ###Biofuels, Food crops | ###Cashew, Corn (Maize), Jatropha, Soya Beans, Sun Flower |
| **3764** | **Africa** | **Ghana** | 2220 | 2220 | 2220 | ###Food crops | ###Rice |
| **3765** | **Africa** | **Ghana** | 3500 | 3500 | 1500 | ###Food crops | ###Banana, Pineapple |
| **3768** | **Africa** | **Ghana** | 1000 | 1000 | 300 | ###Food crops | ###Rice |
| **3770** | **Africa** | **Ghana** | 3750 | 3750 | 2050 | ###Agriculture unspecified | ###Oil Palm |
| **3773** | **Africa** | **Ghana** | 5018 | 5018 | 2000 | ###Agriculture unspecified | ###Oil Palm |
| **3780** | **Africa** | **Swaziland** | 31584 | 31584 | 0 | ###Timber plantation (for wood and fibre) | ###Eucalyptus, Pine |
| **3783** | **Africa** | **Zambia** | 38760 | 38760 | 3000 | ###Food crops, Livestock | ###Barley, Corn (Maize), Potatoes, Soya Beans, Wheat |
| **3800** | **Europe** | **Russian Federation** | 80000 | 80000 | 15000 | ###Food crops | ###Cereals (unspecified) |
| **3819** | **Africa** | **Ethiopia** | 450 | 450 | 120 | ###Food crops | ###Grapes |
| **3846** | **Asia** | **Indonesia** | 2961 | 2961 | 2856 | ###Agriculture unspecified | ###Oil Palm |
| **3847** | **Asia** | **Indonesia** | 1813 | 1813 | 1681 | ###Agriculture unspecified | ###Oil Palm |
| **3859** | **Asia** | **Indonesia** | 18600 | 18600 | 2527 | ###Agriculture unspecified | ###Oil Palm |
| **3866** | **Africa** | **Congo, Dem. Rep.** | 10000 | 10000 | 0 | ###Food crops, Timber plantation (for wood and fibre) | ###Corn (Maize), Eucalyptus, Oil Palm, Rice |
| **3891** | **South America** | **Colombia** | 50000 | 50000 | 38096 | 2017##2027#Biofuels\|2017##80#Food crops\|2017##790#Fodder\|2014##662#Timber plantation (for wood and fibre) | 2017##2027#Oil Palm\|2014##644#Accacia\|2017##80#Corn (Maize), Soya Beans\|##18#Eucalyptus |
| **3901** | **Africa** | **Liberia** | 119344 | 119344 | 0 | ###Timber plantation (for wood and fibre) | ###Trees (unspecified) |
| **3915** | **Africa** | **Ghana** | 405 | 405 | 0 | ###Food crops | ###Cacao, Corn (Maize), Pineapple |
| **3986** | **Africa** | **Sudan** | 87200 | 87200 | 3150 | ###Food crops, Non-food agricultural commodities | ###Sun Flower, Sesame, Alfalfa, Corn (Maize) |
| **4055** | **Africa** | **Senegal** | 4500 | 4500 | 215 | ###Food crops | ###Rice |
| **4059** | **Africa** | **Senegal** | 2750 | 2750 | 574 | ###Biofuels, For carbon sequestration/REDD | ###Peanut (groundnut), Jatropha |
| **4060** | **Africa** | **Zambia** | 440 | 440 | 440 | ###Biofuels, Food crops, Renewable Energy | ###Sugar Cane |
| **4117** | **Asia** | **Philippines** | 300 | 300 | 0 | ###Biofuels | ###Jatropha |
| **4171** | **South America** | **Brazil** | 10853 | 10853 | 8637 | ###Food crops | ###Cotton, Soya Beans |
| **4218** | **South America** | **Brazil** | 3529 | 3529 | 2800 | ###Food crops | ###Cotton, Corn (Maize), Soya Beans |
| **4219** | **South America** | **Brazil** | 1440 | 1440 | 1440 | ###Food crops | ###Papaya, Coffee Plant, Grains (unspecified) |
| **4287** | **Africa** | **Zambia** | 1575 | 1575 | 148 | ###Food crops | ###Barley, Corn (Maize), Sorghum, Soya Beans, Wheat |
| **4341** | **Africa** | **Ghana** | 5223 | 5223 | 0 | ###Food crops | ###Corn (Maize), Rice |
| **4389** | **Africa** | **Botswana** | 25074 | 25074 | 19385 | ###Food crops, Livestock, Non-food agricultural commodities, Agriculture unspecified | ###Onion, Mango, Papaya, Citrus Fruits (unspecified), Cotton, Sorghum, Sun Flower, Banana, Bean, Fruit (unspecified), Grains (unspecified), Potatoes, Soya Beans, Vegetables (unspecified), Wheat |
| **4411** | **Europe** | **Romania** | 9998 | 9998 | 6267 | ###Food crops | ###Sun Flower, Corn (Maize), Grains (unspecified) |
| **4412** | **Europe** | **Romania** | 3000 | 3000 | 0 | ###Food crops, Agriculture unspecified | ###Sun Flower, Soya Beans, Wheat |
| **4413** | **Europe** | **Romania** | 2700 | 2700 | 5500 | ###Food crops, Agriculture unspecified | ###Sorghum, Sun Flower, Corn (Maize), Wheat |
| **4414** | **Europe** | **Romania** | 3000 | 3000 | 3000 | ###Food crops | ###Sun Flower, Canola, Corn (Maize), Soya Beans, Wheat |

| Land Matrix ID | Target country | Target region | Intended size [ha] | Contracted size [ha] | In prod. size [ha] | Intention of investment | Crops |
|---|---|---|---|---|---|---|---|
| **4415** | **Europe** | **Romania** | 1200 | 1200 | 0 | ###Food crops | ###Sun Flower, Canola, Corn (Maize), Soya Beans, Wheat |
| **4417** | **Europe** | **Romania** | 2500 | 2500 | 0 | ###Food crops, Agriculture unspecified | ###Sun Flower, Canola, Corn (Maize), Soya Beans, Wheat |
| **4419** | **Europe** | **Romania** | 8377 | 8377 | 0 | ###Food crops, Livestock | ###Sun Flower, Canola, Corn (Maize), Soya Beans, Wheat |
| **4433** | **Africa** | **Malawi** | 6159 | 6159 | 6159 | ###Food crops | ###Sugar Cane |
| **4470** | **Asia** | **Indonesia** | 12800 | 12800 | 4982 | ###Agriculture unspecified | ###Oil Palm |
| **4493** | **Africa** | **Mozambique** | 500 | 500 | 500 | ###Livestock | 0 |
| **4503** | **Europe** | **Romania** | 3256 | 3256 | 2456 | ###Food crops | ###Barley, Sun Flower, Corn (Maize), Wheat |
| **4509** | **Europe** | **Romania** | 12000 | 12000 | 10170 | ###Food crops, Livestock, Non-food agricultural commodities | ###Barley, Sun Flower, Rapeseed, Rye, Corn (Maize), Wheat |
| **4513** | **Europe** | **Romania** | 4800 | 4800 | 4620 | ###Food crops, Agriculture unspecified | ###Sun Flower, Vineyard, Corn (Maize), Soya Beans, Wheat |
| **4527** | **Africa** | **Egypt, Arab Rep.** | 9510 | 9510 | 9510 | ###Food crops | ###Corn (Maize), Potatoes, Wheat |
| **4557** | **Asia** | **Mongolia** | 270 | 270 | 170 | ###Food crops, Livestock, Tourism, Conservation | ###Fodder Plants (unspecified), Barley, Grains (unspecified), Wheat |
| **4563** | **Europe** | **Romania** | 11000 | 11000 | 10500 | ###Food crops | ###Rice |
| **4582** | **Africa** | **Ghana** | 1000 | 1000 | 0 | ###Food crops, Non-food agricultural commodities, Agriculture unspecified | ###Cashew, Oil Palm |
| **4583** | **Africa** | **Ghana** | 500 | 500 | 400 | ###Food crops | ###Mango, Papaya, Pineapple |
| **4594** | **Europe** | **Ukraine** | 3000 | 3000 | 0 | ###Food crops, Agriculture unspecified | ###Barley, Rapeseed, Peas, Alfalfa, Corn (Maize), Soya Beans, Wheat |
| **4616** | **Europe** | **Ukraine** | 11000 | 11000 | 0 | ###Food crops, Livestock, Agriculture unspecified | ###Barley, Corn (Maize), Soya Beans, Sun Flower, Wheat |
| **4618** | **Europe** | **Ukraine** | 7400 | 0 | 7400 | ###Food crops, Livestock, Agriculture unspecified | ###Vegetables (unspecified) |
| **4730** | **Africa** | **Ghana** | 650 | 650 | 0 | ###Agriculture unspecified | ###Oil Palm |
| **4732** | **Europe** | **Bulgaria** | 2000 | 2000 | 0 | ###Food crops | ###Sun Flower, Alfalfa, Corn (Maize) |
| **4745** | **Africa** | **Uganda** | 6030 | 6030 | 1700 | ###Food crops, Agriculture unspecified | ###Corn (Maize), Rice, Sorghum, Soya Beans, Sun Flower |
| **4802** | **Asia** | **Indonesia** | 8374 | 8374 | 0 | ###Agriculture unspecified | ###Oil Palm |
| **4924** | **Africa** | **Zimbabwe** | 10000 | 10000 | 0 | ###Food crops | ###Corn (Maize), Soya Beans, Wheat |
| **4929** | **Africa** | **Sierra Leone** | 2500 | 2500 | 1330 | ###Biofuels, Food crops, Agriculture unspecified | ###Cacao, Coffee Plant, Oil Palm, Rubber tree |
| **4930** | **Africa** | **Senegal** | 310 | 310 | 310 | ###Food crops | ###Onion, Fruit (unspecified), Potatoes, Vegetables (unspecified) |
| **4931** | **Africa** | **Senegal** | 5700 | 5700 | 346 | ###Food crops | ###Onion, Papaya, Seeds Production (unspecified), Banana, Corn (Maize), Potatoes, Rice, Wheat |
| **4940** | **Africa** | **Ghana** | 5740 | 5740 | 365 | ###Food crops | ###Onion, Sorghum, Corn (Maize), Rice, Soya Beans |
| **5024** | **Africa** | **Uganda** | 12182 | 12182 | 2624 | ###Timber plantation (for wood and fibre), For carbon sequestration/REDD | ###Eucalyptus, Pine, Trees (unspecified) |
| **5316** | **Africa** | **Ghana** | 635 | 635 | 405 | ###Food crops, Conservation | ###Mango |
| **5385** | **Europe** | **Serbia** | 699 | 699 | 699 | ###Food crops | ###Other crops (please specify) |
| **5389** | **Europe** | **Ukraine** | 12300 | 12300 | 12300 | ###Food crops, Livestock | ###Sun Flower, Peas, Mustard, Corn (Maize), Soya Beans, Wheat |
| **5501** | **Europe** | **Russian Federation** | 46900 | 46900 | 0 | ###Food crops, Livestock | ###Grains (unspecified) |
| **5519** | **Europe** | **Ukraine** | 1500 | 1500 | 0 | ###Food crops | ###Sugar beet |
| **5551** | **Europe** | **Romania** | 9800 | 9800 | 0 | ###Biofuels, Food crops | ###Barley, Sun Flower, Rapeseed, Corn (Maize), Wheat |
| **5585** | **Africa** | **Cameroon** | 114379 | 114379 | 0 | ###Timber plantation (for wood and fibre) | ###Trees (unspecified) |
| **5586** | **Africa** | **Cameroon** | 195921 | 195921 | 0 | ###Timber plantation (for wood and fibre) | ###Trees (unspecified) |
| **5850** | **Asia** | **Myanmar** | 15378 | 15378 | 2428 | ###Agriculture unspecified | ###Oil Palm |
| **5863** | **Asia** | **Myanmar** | 12545 | 12545 | 373 | ###Agriculture unspecified | ###Oil Palm |
| **6057** | **Africa** | **Ghana** | 80000 | 80000 | 20000 | ###Timber plantation (for wood and fibre) | ###Eucalyptus |
| **6079** | **Europe** | **Ukraine** | 15000 | 15000 | 0 | ###Food crops | ###Barley, Corn (Maize), Soya Beans, Sun Flower, Wheat |
| **6086** | **Europe** | **Ukraine** | 1700 | 1700 | 0 | ###Food crops | ###Corn (Maize), Rapeseed, Soya Beans, Sun Flower, Wheat |
| **6087** | **Europe** | **Ukraine** | 700 | 700 | 0 | ###Food crops, Livestock | ###Corn (Maize), Soya Beans, Wheat |

| Land Matrix ID | Target country | Target region | Intended size [ha] | Contracted size [ha] | In prod. size [ha] | Intention of investment | Crops |
|---|---|---|---|---|---|---|---|
| **6096** | **Europe** | **Romania** | 4000 | 4000 | 0 | ###Timber plantation (for wood and fibre), Renewable Energy | ###Trees (unspecified) |
| **6097** | **Europe** | **Romania** | 4036 | 4036 | 0 | ###Biofuels | ###Food crops (unspecified), Wheat |
| **6101** | **Europe** | **Russian Federation** | 6200 | 6200 | 3750 | ###Food crops | ###Barley, Corn (Maize), Wheat |
| **6104** | **Europe** | **Ukraine** | 6400 | 6400 | 0 | ###Food crops | ###Corn (Maize), Soya Beans, Sugar beet, Sun Flower, Wheat |
| **6113** | **Europe** | **Ukraine** | 11400 | 11400 | 0 | ###Food crops | ###Barley, Corn (Maize), Rapeseed, Soya Beans, Sun Flower, Wheat |
| **6114** | **Europe** | **Ukraine** | 2500 | 2500 | 0 | ###Food crops | ###Barley, Corn (Maize), Rapeseed, Soya Beans, Sun Flower, Wheat |
| **6115** | **Europe** | **Ukraine** | 3200 | 3200 | 0 | ###Food crops | ###Barley, Corn (Maize), Rapeseed, Soya Beans, Sun Flower, Wheat |
| **6119** | **Europe** | **Ukraine** | 2400 | 2400 | 0 | ###Food crops, Livestock, Non-food agricultural commodities | ###Barley, Corn (Maize), Soya Beans, Sun Flower, Wheat |
| **6727** | **Asia** | **Indonesia** | 32300 | 32300 | 0 | ###Agriculture unspecified | ###Oil Palm |
| **6777** | **Asia** | **Myanmar** | 54066 | 54066 | 1600 | ###Agriculture unspecified, Forest logging / management (for wood and fibre) | ##1600#Oil Palm |
| **1742** | **Africa** | **South Sudan** | NA | NA | NA | NA | NA |
| **1820** | **Africa** | **Sierra Leone** | NA | NA | NA | NA | NA |
| **3037** | **Africa** | **Mozambique** | NA | NA | NA | NA | NA |
| **3038** | **Africa** | **Mozambique** | NA | NA | NA | NA | NA |
| **3458** | **Africa** | **Liberia** | NA | NA | NA | NA | NA |
| **4418** | **Europe** | **Romania** | NA | NA | NA | NA | NA |
| **4576** | **Europe** | **Romania** | NA | NA | NA | NA | NA |
| **5600** | **Africa** | **Mozambique** | NA | NA | NA | NA | NA |
| **5883** | **South America** | **Nicaragua** | NA | NA | NA | NA | NA |

**Supplementary table S2.** River distance and mean groundwater table depth

| Deal_ID | Distance from river [m] | MEAN Groundwater_Depth [m] |
|---|---|---|
| **23** | 29820.3 | 31.6 |
| **33** | 15630.5 | 4.1 |
| **35** | 0.0 | 8.2 |
| **42** | 2167.1 | 1.4 |
| **105** | 0.0 | 1.2 |
| **138** | 8416.8 | 1.8 |
| **427** | 0.0 | 0.9 |
| **614** | 31033.4 | 4.8 |
| **746** | 0.0 | 4.1 |
| **767** | 0.0 | 18.3 |
| **877** | 20225.8 | 4.0 |
| **1037** | 0.0 | 12.8 |
| **1064** | 0.0 | 0.2 |
| **1065** | 0.0 | 0.1 |
| **1066** | 7086.0 | 6.9 |
| **1067** | 0.0 | 0.4 |
| **1113** | 0.0 | 12.2 |
| **1141** | 0.0 | 5.4 |
| **1166** | 0.0 | 0.3 |

| Deal_ID | Distance from river [m] | MEAN Groundwater_Depth [m] |
|---|---|---|
| **1173** | 0.0 | 4.7 |
| **1205** | 0.0 | 0.9 |
| **1218** | 16617.6 | 58.7 |
| **1244** | 0.0 | 3.4 |
| **1322** | 0.0 | 16.0 |
| **1323** | 0.0 | 3.6 |
| **1388** | 0.0 | 0.9 |
| **1467** | 0.0 | 27.4 |
| **1562** | 0.0 | 0.1 |
| **1575** | 0.0 | 40.7 |
| **1652** | 0.0 | 2.3 |
| **1684** | 0.0 | 2.2 |
| **1721** | 0.0 | 0.9 |
| **1777** | 0.0 | 1.8 |
| **1795** | 0.0 | 2.4 |
| **1798** | 0.0 | 0.2 |
| **1817** | 1024.4 | 4.8 |
| **1839** | 4637.7 | 0.0 |
| **1976** | 12300.2 | 12.2 |
| **2024** | 6989.6 | 13.0 |
| **2053** | 4564.0 | 8.7 |
| **2241** | 0.0 | 10.6 |
| **2371** | 1234.3 | 0.1 |
| **2385** | 86.1 | 1.7 |
| **3008** | 0.0 | 0.8 |
| **3031** | 770.6 | 5.9 |
| **3272** | 11911.5 | 11.3 |
| **3389** | 22364.3 | 6.5 |
| **3393** | 0.0 | 1.3 |
| **3398** | 0.0 | 8.0 |
| **3399** | 0.0 | 3.3 |
| **3403** | 0.0 | 16.8 |
| **3404** | 0.0 | 14.8 |
| **3421** | 0.0 | 2.9 |
| **3422** | 2678.5 | 6.8 |
| **3459** | 0.0 | 4.3 |
| **3462** | 0.0 | 5.3 |
| **3463** | 10765.9 | 4.6 |
| **3470** | 1949.2 | 1.5 |
| **3472** | 2.2 | 2.6 |
| **3479** | 0.0 | 2.5 |
| **3484** | 2.2 | 28.0 |
| **3486** | 779.9 | 1.2 |
| **3503** | 0.0 | 0.0 |
| **3510** | 6460.2 | 9.9 |
| **3525** | 0.0 | 3.0 |

| Deal_ID | Distance from river [m] | MEAN Groundwater_Depth [m] |
|---|---|---|
| **3527** | 13773.1 | 2.1 |
| **3533** | 0.0 | 0.0 |
| **3534** | 0.0 | 1.1 |
| **3539** | 5545.0 | 0.0 |
| **3554** | 0.0 | 1.1 |
| **3593** | 0.0 | 0.5 |
| **3612** | 7589.1 | 79.9 |
| **3614** | 5770.3 | 0.4 |
| **3618** | 0.0 | 1.0 |
| **3640** | 0.0 | 3.3 |
| **3641** | 0.0 | 4.6 |
| **3715** | 15327.8 | 0.0 |
| **3761** | 20745.5 | 19.3 |
| **3764** | 0.0 | 0.8 |
| **3765** | 3260.1 | 42.6 |
| **3768** | 0.0 | 5.5 |
| **3770** | 0.0 | 13.6 |
| **3773** | 3865.5 | 4.2 |
| **3780** | 0.0 | 65.3 |
| **3783** | 14594.7 | 1.0 |
| **3800** | 0.0 | 2.1 |
| **3819** | 0.0 | 0.4 |
| **3846** | 0.0 | 0.2 |
| **3847** | 0.0 | 0.2 |
| **3859** | 0.0 | 1.0 |
| **3866** | 3123.5 | 0.0 |
| **3891** | 0.0 | 5.1 |
| **3901** | 1556.7 | 12.8 |
| **3915** | 0.0 | 10.0 |
| **3986** | 6616.5 | 0.4 |
| **4055** | 20644.3 | 2.8 |
| **4059** | 8098.6 | 47.1 |
| **4060** | 57355.8 | 0.0 |
| **4117** | 29627.6 | 6.9 |
| **4171** | 31856.8 | 13.5 |
| **4218** | 9862.2 | 3.6 |
| **4219** | 0.0 | 0.1 |
| **4287** | 0.0 | 0.4 |
| **4341** | 0.0 | 6.3 |
| **4389** | 0.0 | 0.8 |
| **4411** | 9130.6 | 80.7 |
| **4412** | 6263.4 | 55.6 |
| **4413** | 0.0 | 42.3 |
| **4414** | 0.0 | 0.9 |
| **4415** | 20333.2 | 2.7 |
| **4417** | 2698.9 | 16.5 |

| Deal_ID | Distance from river [m] | MEAN Groundwater_Depth [m] |
|---|---|---|
| **4419** | 2596.7 | 0.0 |
| **4433** | 0.0 | 0.6 |
| **4470** | 15957.4 | 0.1 |
| **4493** | 10224.5 | 3.0 |
| **4503** | 3139.7 | 0.3 |
| **4509** | 12634.7 | 6.7 |
| **4513** | 0.0 | 1.9 |
| **4527** | 4043.5 | 4.0 |
| **4557** | 0.0 | 0.1 |
| **4563** | 0.0 | 4.7 |
| **4582** | 5222.3 | 10.8 |
| **4583** | 0.0 | 62.6 |
| **4594** | 15339.6 | 28.9 |
| **4616** | 0.0 | 9.4 |
| **4618** | 3134.8 | 2.6 |
| **4730** | 755.8 | 26.3 |
| **4732** | 13957.2 | 61.5 |
| **4745** | 0.0 | 8.2 |
| **4802** | 2359.1 | 3.7 |
| **4924** | 0.0 | 23.7 |
| **4929** | 8378.0 | 3.0 |
| **4930** | 9551.7 | 3.5 |
| **4931** | 0.0 | 10.7 |
| **4940** | 0.0 | 4.7 |
| **5024** | 0.0 | 0.0 |
| **5316** | 828.2 | 0.1 |
| **5385** | 497.5 | 1.2 |
| **5389** | 18156.9 | 17.7 |
| **5501** | 0.0 | 17.9 |
| **5519** | 0.0 | 0.0 |
| **5551** | 0.0 | 17.2 |
| **5585** | 0.0 | 11.4 |
| **5586** | 0.0 | 32.4 |
| **5850** | 0.0 | 34.5 |
| **5863** | 991.5 | 22.4 |
| **6057** | 149.0 | 25.4 |
| **6079** | 347.2 | 4.4 |
| **6086** | 0.0 | 12.2 |
| **6087** | 14506.3 | 4.8 |
| **6096** | 1387.0 | 41.4 |
| **6097** | 740.6 | 0.2 |
| **6101** | 5181.7 | 34.4 |
| **6104** | 10833.4 | 34.4 |
| **6113** | 16581.9 | 34.8 |
| **6114** | 14891.7 | 38.2 |
| **6115** | 7194.5 | 32.6 |

| Deal_ID | Distance from river [m] | MEAN Groundwater_Depth [m] |
|---|---|---|
| **6119** | 0.0 | 17.9 |
| **6727** | 0.0 | 1.1 |
| **6777** | 0.0 | 5.9 |
| **Average** | 4479.6 | 11.0 |

**Supplementary table S3.** Blue water volume [m3] used before large-scale land deals (Before acquisition scenario. i.e.. area and crops cultivated before the acquisition). in the current scenario (Current use scenario. i.e.. current cultivated area and intended crops) and under potential cultivation expansion (100% cultivated scenario. i.e.. entire acquired area cultivated with intended crops).

| Deal_ID | Target region | Target country | Scenario Before Acquisition [m3] | Scenario Current Use [m3] | Scenario 100% cultivated [m3] |
|---|---|---|---|---|---|
| 23 | Asia | Cambodia | 65838 | 0 | 0 |
| 33 | Asia | Cambodia | 41828 | 0 | 0 |
| 35 | Asia | Cambodia | 374226 | 0 | 0 |
| 42 | Asia | Cambodia | 0 | 0 | 0 |
| 105 | Asia | Cambodia | 0 | 0 | 0 |
| 138 | Asia | China | 304451 | 108978 | 180498 |
| 138 | Asia | China | 18332 | 0 | 0 |
| 427 | Asia | Philippines | 80087 | 0 | 0 |
| 614 | Asia | India | 459357 | 0 | 0 |
| 614 | Asia | India | 1367529 | 0 | 0 |
| 746 | South America | Brazil | 44496 | 479013271 | 725938890 |
| 767 | South America | Brazil | 0 | 0 | 0 |
| 877 | South America | Paraguay | 75203 | 420863 | 2867738 |
| 1037 | Europe | Ukraine | 0 | 129435370 | 164832691 |
| 1037 | Europe | Ukraine | 0 | 218276927 | 277970180 |
| 1064 | South America | Argentina | 1620201 | 38269765 | 89438930 |
| 1065 | South America | Argentina | 0 | 74843246 | 103038884 |
| 1066 | South America | Argentina | 0 | 55670239 | 62186324 |
| 1067 | South America | Argentina | 0 | 77912256 | 97248445 |
| 1113 | Africa | Benin | 205801 | 9486890 | 16453570 |
| 1141 | Africa | Cameroon | 0 | 5837203 | 7342891 |
| 1141 | Africa | Cameroon | 0 | 5466987 | 6877179 |
| 1166 | Africa | Congo. Rep. | 0 | 2648126 | 221031577 |
| 1166 | Africa | Congo. Rep. | 0 | 2741459 | 228821850 |
| 1173 | Africa | Egypt | 0 | 104427796 | 296744667 |
| 1205 | Africa | Ethiopia | 0 | 9965500 | 35047091 |
| 1218 | Africa | Ethiopia | 0 | 907049 | 995630 |
| 1244 | Africa | Ethiopia | 0 | 238737 | 18725683 |
| 1322 | Africa | Ghana | 0 | 2263808 | 41274833 |
| 1323 | Africa | Ghana | 49260 | 0 | 0 |
| 1388 | Africa | Liberia | 0 | 7403258 | 622487580 |
| 1467 | Africa | Mali | 0 | 86730869 | 228689600 |
| 1562 | Africa | Mozambique | 0 | 66979 | 5631768 |

| Deal_ID | Target region | Target country | Scenario Before Acquisition [m3] | Scenario Current Use [m3] | Scenario 100% cultivated [m3] |
|---|---|---|---|---|---|
| 1575 | Africa | Mozambique | 0 | 4826987 | 49852509 |
| 1652 | Africa | Namibia | 0 | 533918 | 1874418 |
| 1684 | Africa | Nigeria | 0 | 7621975 | 46612260 |
| 1721 | Africa | Sudan | 0 | 14682663 | 220002180 |
| 1777 | Africa | Senegal | 1804752 | 1635742 | 2936695 |
| 1795 | Africa | Senegal | 0 | 0 | 0 |
| 1795 | Africa | Senegal | 0 | 255649 | 3275889 |
| 1795 | Africa | Senegal | 0 | 265065 | 3396539 |
| 1798 | Africa | Sierra Leone | 2100383 | 5989301 | 166109139 |
| 1817 | Africa | Sierra Leone | 0 | 879661 | 4029411 |
| 1839 | Africa | Tanzania | 1499246 | 31305947 | 42225805 |
| 1976 | Africa | Uganda | 0 | 1093531 | 3737673 |
| 2024 | Africa | Zambia | 0 | 17157442 | 146002442 |
| 2053 | Africa | Zambia | 161602 | 7760114 | 21675400 |
| 2241 | Africa | Ghana | 0 | 1441586 | 20291079 |
| 2371 | Africa | Sierra Leone | 0 | 986239 | 82925903 |
| 2385 | Africa | Mozambique | 12611 | 3457144 | 3939096 |
| 3008 | Africa | Sierra Leone | 26895 | 6454393 | 10601204 |
| 3031 | Africa | Mozambique | 0 | 26302123 | 69848837 |
| 3272 | Africa | Cost D’Ivoire | 80453 | 11751999 | 15919515 |
| 3389 | Africa | Ghana | 0 | 0 | 0 |
| 3393 | Africa | Ghana | 0 | 33409870 | 1400771733 |
| 3398 | Africa | Ghana | 0 | 9817635 | 305179500 |
| 3399 | Africa | Ghana | 0 | 0 | 0 |
| 3403 | Africa | Congo. Rep. | 0 | 0 | 0 |
| 3404 | Africa | Ghana | 0 | 0 | 0 |
| 3421 | Asia | Cambodia | 0 | 0 | 0 |
| 3422 | Asia | Cambodia | 0 | 0 | 0 |
| 3459 | Asia | Cambodia | 0 | 0 | 0 |
| 3462 | Asia | Cambodia | 0 | 0 | 0 |
| 3463 | Africa | Liberia | 0 | 25503 | 2144402 |
| 3470 | Asia | Cambodia | 3852 | 0 | 0 |
| 3472 | Asia | Cambodia | 0 | 0 | 0 |
| 3479 | Asia | Cambodia | 0 | 0 | 0 |
| 3484 | Asia | Cambodia | 1526 | 0 | 0 |
| 3486 | Asia | Cambodia | 0 | 2116203 | 2534530 |
| 3503 | Asia | Cambodia | 1097976 | 0 | 0 |
| 3510 | Asia | Cambodia | 0 | 0 | 0 |
| 3525 | Asia | Cambodia | 22465 | 11215741 | 36105358 |
| 3527 | Asia | Cambodia | 41490 | 0 | 0 |
| 3533 | Asia | Cambodia | 6059 | 15603533 | 30718685 |
| 3534 | Asia | Cambodia | 0 | 0 | 0 |
| 3539 | Asia | Cambodia | 0 | 0 | 0 |
| 3554 | Asia | Cambodia | 0 | 0 | 0 |
| 3593 | Asia | China | 0 | 67160895 | 280130768 |

| Deal_ID | Target region | Target country | Scenario Before Acquisition [m3] | Scenario Current Use [m3] | Scenario 100% cultivated [m3] |
|---|---|---|---|---|---|
| 3612 | Asia | Indonesia | 0 | 11421 | 487177 |
| 3614 | Asia | Indonesia | 0 | 442035 | 908332 |
| 3618 | Asia | Indonesia | 0 | 0 | 0 |
| 3640 | Asia | Indonesia | 0 | 560080 | 957802 |
| 3640 | Asia | Indonesia | 0 | 0 | 0 |
| 3641 | Asia | Indonesia | 0 | 0 | 0 |
| 3641 | Asia | Indonesia | 0 | 14456 | 51854 |
| 3715 | Asia | Indonesia | 319774 | 0 | 0 |
| 3761 | Africa | Ghana | 0 | 12251 | 1030072 |
| 3764 | Africa | Ghana | 0 | 148818 | 5647345 |
| 3765 | Africa | Ghana | 4785 | 1659095 | 3056655 |
| 3765 | Africa | Ghana | 6053 | 2118541 | 3903121 |
| 3768 | Africa | Ghana | 0 | 1388563 | 2234261 |
| 3770 | Africa | Ghana | 0 | 2104424 | 12730031 |
| 3770 | Africa | Ghana | 0 | 1049027 | 6345750 |
| 3773 | Africa | Ghana | 0 | 17646 | 1483722 |
| 3773 | Africa | Ghana | 0 | 125888 | 10585012 |
| 3780 | Africa | Swaziland | 0 | 0 | 0 |
| 3783 | Africa | Zambia | 0 | 15738521 | 87363521 |
| 3800 | Europe | Russian Federation | 0 | 0 | 0 |
| 3819 | Africa | Ethiopia | 14093 | 86877 | 134033 |
| 3846 | Asia | Indonesia | 0 | 0 | 0 |
| 3847 | Asia | Indonesia | 0 | 0 | 0 |
| 3859 | Asia | Indonesia | 1101286 | 328647 | 552680 |
| 3866 | Africa | Congo. Dem. Rep. | 0 | 1596966 | 4978098 |
| 3891 | South America | Colombia | 0 | 6007776 | 51408250 |
| 3901 | Africa | Liberia | 0 | 0 | 0 |
| 3915 | Africa | Ghana | 0 | 11182 | 940250 |
| 3986 | Africa | Sudan | 0 | 87967250 | 673020936 |
| 4055 | Africa | Senegal | 423369 | 5608071 | 24464430 |
| 4059 | Africa | Senegal | 0 | 9373003 | 11743671 |
| 4059 | Africa | Senegal | 0 | 11326744 | 14191561 |
| 4060 | Africa | Zambia | 113372 | 61902 | 68957 |
| 4117 | Asia | Philippines | 0 | 0 | 0 |
| 4171 | South America | Brazil | 0 | 9898918 | 27933359 |
| 4218 | South America | Brazil | 570516 | 13577237 | 14636168 |
| 4219 | South America | Brazil | 40981 | 1619646 | 2275474 |
| 4287 | Africa | Zambia | 177133 | 51927 | 4224033 |
| 4341 | Africa | Ghana | 0 | 47940 | 1547967 |
| 4389 | Africa | Botswana | 0 | 18711418 | 53714198 |
| 4389 | Africa | Botswana | 0 | 22803807 | 65462071 |
| 4411 | Europe | Romania | 3287461 | 14294909 | 19859234 |
| 4412 | Europe | Romania | 23067 | 695201 | 998495 |
| 4412 | Europe | Romania | 35077 | 1056647 | 1517627 |

| Deal_ID | Target region | Target country | Scenario Before Acquisition [m3] | Scenario Current Use [m3] | Scenario 100% cultivated [m3] |
|---|---|---|---|---|---|
| 4412 | Europe | Romania | 35825 | 1072927 | 1541009 |
| 4413 | Europe | Romania | 46084 | 3178698 | 3506795 |
| 4414 | Europe | Romania | 0 | 4766359 | 5153093 |
| 4415 | Europe | Romania | 0 | 464947 | 493043 |
| 4417 | Europe | Romania | 0 | 2021959 | 2084966 |
| 4419 | Europe | Romania | 0 | 4265981 | 4796327 |
| 4419 | Europe | Romania | 0 | 3098450 | 3483649 |
| 4419 | Europe | Romania | 0 | 3098450 | 3483649 |
| 4433 | Africa | Malawi | 11519739 | 15580113 | 20964026 |
| 4433 | Africa | Malawi | 5890221 | 7819059 | 10521038 |
| 4470 | Asia | Indonesia | 82447 | 0 | 0 |
| 4493 | Africa | Mozambique | 0 | 7162 | 602161 |
| 4503 | Europe | Romania | 67561 | 3038788 | 3152270 |
| 4509 | Europe | Romania | 0 | 3194416 | 3296771 |
| 4509 | Europe | Romania | 0 | 5163518 | 5328966 |
| 4509 | Europe | Romania | 0 | 4742298 | 4894249 |
| 4513 | Europe | Romania | 69997 | 909294 | 1475572 |
| 4513 | Europe | Romania | 168033 | 2218111 | 3599475 |
| 4513 | Europe | Romania | 94367 | 1214400 | 1970687 |
| 4527 | Africa | Egypt | 0 | 43032880 | 109101605 |
| 4557 | Asia | Mongolia | 0 | 4928 | 210191 |
| 4563 | Europe | Romania | 577288 | 16027670 | 28435204 |
| 4563 | Europe | Romania | 223757 | 7954237 | 14111867 |
| 4582 | Africa | Ghana | 0 | 17641 | 1483309 |
| 4583 | Africa | Ghana | 0 | 471473 | 537721 |
| 4594 | Europe | Ukraine | 0 | 495268 | 890901 |
| 4594 | Europe | Ukraine | 0 | 554849 | 998077 |
| 4594 | Europe | Ukraine | 0 | 387344 | 696764 |
| 4616 | Europe | Ukraine | 887374 | 2798107 | 3954493 |
| 4618 | Europe | Ukraine | 0 | 0 | 0 |
| 4618 | Europe | Ukraine | 0 | 0 | 0 |
| 4730 | Africa | Ghana | 0 | 52485 | 4413078 |
| 4732 | Europe | Bulgaria | 0 | 1009425 | 1192992 |
| 4745 | Africa | Uganda | 0 | 525843 | 9078397 |
| 4802 | Asia | Indonesia | 0 | 217971 | 1137704 |
| 4802 | Asia | Indonesia | 0 | 216047 | 1127663 |
| 4924 | Africa | Zimbabwe | 2296472 | 21031541 | 55034367 |
| 4929 | Africa | Sierra Leone | 2778203 | 126173 | 10609000 |
| 4930 | Africa | Senegal | 0 | 587076 | 1496104 |
| 4931 | Africa | Senegal | 0 | 6557903 | 35946686 |
| 4940 | Africa | Ghana | 0 | 102771 | 5976731 |
| 5024 | Africa | Uganda | 0 | 0 | 0 |
| 5316 | Africa | Ghana | 0 | 1329 | 111785 |
| 5385 | Europe | Serbia | 1534 | 0 | 0 |
| 5389 | Europe | Ukraine | 33808 | 1071639 | 1513782 |

| Deal_ID | Target region | Target country | Scenario Before Acquisition [m3] | Scenario Current Use [m3] | Scenario 100% cultivated [m3] |
|---|---|---|---|---|---|
| 5389 | Europe | Ukraine | 72037 | 1496209 | 2113524 |
| 5389 | Europe | Ukraine | 44988 | 282984 | 399740 |
| 5389 | Europe | Ukraine | 40365 | 476010 | 672405 |
| 5501 | Europe | Russian Federation | 0 | 146334437 | 157488699 |
| 5519 | Europe | Ukraine | 1008678 | 1299562 | 3167805 |
| 5551 | Europe | Romania | 836777 | 7788197 | 10214868 |
| 5585 | Africa | Cameroon | 0 | 0 | 0 |
| 5586 | Africa | Cameroon | 0 | 0 | 0 |
| 5850 | Asia | Myanmar | 0 | 4540472 | 51930291 |
| 5863 | Asia | Myanmar | 0 | 3157328 | 30371320 |
| 6057 | Africa | Ghana | 0 | 0 | 0 |
| 6079 | Europe | Ukraine | 175747 | 7964699 | 9103878 |
| 6079 | Europe | Ukraine | 94004 | 5482008 | 6266091 |
| 6086 | Europe | Ukraine | 176234 | 341502 | 398971 |
| 6087 | Europe | Ukraine | 0 | 332482 | 419060 |
| 6096 | Europe | Romania | 0 | 0 | 0 |
| 6096 | Europe | Romania | 0 | 0 | 0 |
| 6097 | Europe | Romania | 0 | 4638179 | 4809143 |
| 6101 | Europe | Russia | 76065 | 620827 | 894712 |
| 6104 | Europe | Ukraine | 0 | 494721 | 604598 |
| 6104 | Europe | Ukraine | 0 | 666830 | 814931 |
| 6113 | Europe | Ukraine | 0 | 0 | 0 |
| 6113 | Europe | Ukraine | 0 | 3601561 | 4569707 |
| 6114 | Europe | Ukraine | 0 | 638130 | 858118 |
| 6115 | Europe | Ukraine | 0 | 1011752 | 1141052 |
| 6119 | Europe | Ukraine | 0 | 0 | 0 |
| 6727 | Asia | Indonesia | 0 | 0 | 0 |
| 6777 | Asia | Myanmar | 0 | 8564417 | 144583243 |
| **Total** | | | **44980493** | **2195235107** | **8136351931** |

**Table S4** Blue water withdrawal in the 100% cultivated scenario

| **Deal _ID** | **Target region** | **Target country** | **Scenario 100% cultivated - current irrigation efficiency [m3]** | **Scenario 100% cultivated - irrigation efficiency 75% [m3]** |
|---|---|---|---|---|
| 23 | Asia | Cambodia | 0 | 0 |
| 33 | Asia | Cambodia | 0 | 0 |
| 35 | Asia | Cambodia | 0 | 0 |
| 42 | Asia | Cambodia | 0 | 0 |
| 105 | Asia | Cambodia | 0 | 0 |
| 138 | Asia | China | 564056 | 240664 |
| 138 | Asia | China | 0 | 0 |
| 427 | Asia | Philippines | 0 | 0 |
| 614 | Asia | India | 0 | 0 |
| 614 | Asia | India | 0 | 0 |
| 746 | South America | Brazil | 2022739941 | 967918520 |
| 767 | South America | Brazil | 0 | 0 |
| 877 | South America | Paraguay | 8193538 | 3823651 |
| 1037 | Europe | Ukraine | 459083091 | 219776921 |
| 1037 | Europe | Ukraine | 774187504 | 370626906 |
| 1064 | South America | Argentina | 337505397 | 119251907 |
| 1065 | South America | Argentina | 981322709 | 137385179 |
| 1066 | South America | Argentina | 194332264 | 82915099 |
| 1067 | South America | Argentina | 388993779 | 129664593 |
| 1113 | Africa | Benin | 39175166 | 21938093 |
| 1141 | Africa | Cameroon | 26224609 | 9790521 |
| 1141 | Africa | Cameroon | 24561353 | 9169572 |
| 1166 | Africa | Congo. Rep. | 690723677 | 294708769 |
| 1166 | Africa | Congo. Rep. | 715068281 | 305095800 |
| 1173 | Africa | Egypt. Arab Rep. | 1059802381 | 395659556 |
| 1205 | Africa | Ethiopia | 67398252 | 46729455 |
| 1218 | Africa | Ethiopia | 3111345 | 1327507 |
| 1244 | Africa | Ethiopia | 58517760 | 24967578 |
| 1322 | Africa | Ghana | 152869750 | 55033110 |
| 1323 | Africa | Ghana | 0 | 0 |
| 1388 | Africa | Liberia | 1729132167 | 829983440 |
| 1467 | Africa | Mali | 1143448000 | 304919467 |
| 1562 | Africa | Mozambique | 13736020 | 7509024 |
| 1575 | Africa | Mozambique | 109565954 | 66470012 |
| 1652 | Africa | Namibia | 9865356 | 2499223 |
| 1684 | Africa | Nigeria | 221963143 | 62149680 |
| 1721 | Africa | Sudan | 916675751 | 293336240 |
| 1777 | Africa | Senegal | 24472457 | 3915593 |
| 1795 | Africa | Senegal | 0 | 0 |
| 1795 | Africa | Senegal | 26741948 | 4367852 |

| Deal _ID | Target region | Target country | Scenario 100% cultivated - current irrigation efficiency [m3] | Scenario 100% cultivated - irrigation efficiency 75% [m3] |
|---|---|---|---|---|
| 1795 | Africa | Senegal | 27726848 | 4528719 |
| 1798 | Africa | Sierra Leone | 549121121 | 221478852 |
| 1817 | Africa | Sierra Leone | 14923745 | 5372548 |
| 1839 | Africa | Tanzania | 162406941 | 56301073 |
| 1976 | Africa | Uganda | 10101818 | 4983563 |
| 2024 | Africa | Zambia | 608343510 | 194669923 |
| 2053 | Africa | Zambia | 37371380 | 28900534 |
| 2241 | Africa | Ghana | 0 | 27054771 |
| 2371 | Africa | Sierra Leone | 404516602 | 110567871 |
| 2385 | Africa | Mozambique | 12309673 | 5252127 |
| 3008 | Africa | Sierra Leone | 38202537 | 14134939 |
| 3031 | Africa | Mozambique | 170363018 | 93131783 |
| 3272 | Africa | Cote d'Ivoire | 39798786 | 21226019 |
| 3389 | Africa | Ghana | 0 | 0 |
| 3393 | Africa | Ghana | 4002204951 | 1867695644 |
| 3398 | Africa | Ghana | 1130294444 | 406906000 |
| 3399 | Africa | Ghana | 0 | 0 |
| 3403 | Africa | Congo. Rep. | 0 | 0 |
| 3404 | Africa | Ghana | 0 | 0 |
| 3421 | Asia | Cambodia | 0 | 0 |
| 3422 | Asia | Cambodia | 0 | 0 |
| 3459 | Asia | Cambodia | 0 | 0 |
| 3462 | Asia | Cambodia | 0 | 0 |
| 3463 | Africa | Liberia | 5956671 | 2859202 |
| 3470 | Asia | Cambodia | 0 | 0 |
| 3472 | Asia | Cambodia | 0 | 0 |
| 3479 | Asia | Cambodia | 0 | 0 |
| 3484 | Asia | Cambodia | 0 | 0 |
| 3486 | Asia | Cambodia | 9939332 | 3379373 |
| 3503 | Asia | Cambodia | 0 | 0 |
| 3510 | Asia | Cambodia | 0 | 0 |
| 3525 | Asia | Cambodia | 133723549 | 48140478 |
| 3527 | Asia | Cambodia | 0 | 0 |
| 3533 | Asia | Cambodia | 113772906 | 40958246 |
| 3534 | Asia | Cambodia | 0 | 0 |
| 3539 | Asia | Cambodia | 0 | 0 |
| 3554 | Asia | Cambodia | 0 | 0 |
| 3593 | Asia | China | 813282876 | 373507691 |
| 3612 | Asia | Indonesia | 1391933 | 649569 |
| 3614 | Asia | Indonesia | 2595234 | 1211109 |
| 3618 | Asia | Indonesia | 0 | 0 |
| 3640 | Asia | Indonesia | 2902431 | 1277070 |

| Deal _ID | Target region | Target country | Scenario 100% cultivated - current irrigation efficiency [m3] | Scenario 100% cultivated - irrigation efficiency 75% [m3] |
|---|---|---|---|---|
| 3640 | Asia | Indonesia | 0 | 0 |
| 3641 | Asia | Indonesia | 0 | 0 |
| 3641 | Asia | Indonesia | 131608 | 69138 |
| 3715 | Asia | Indonesia | 0 | 0 |
| 3761 | Africa | Ghana | 2943062 | 1373429 |
| 3764 | Africa | Ghana | 16020836 | 7529793 |
| 3765 | Africa | Ghana | 8733300 | 4075540 |
| 3765 | Africa | Ghana | 11151775 | 5204162 |
| 3768 | Africa | Ghana | 6293693 | 2979015 |
| 3770 | Africa | Ghana | 36371518 | 16973375 |
| 3770 | Africa | Ghana | 18130714 | 8461000 |
| 3773 | Africa | Ghana | 4239206 | 1978296 |
| 3773 | Africa | Ghana | 30242891 | 14113349 |
| 3780 | Africa | Swaziland | 0 | 0 |
| 3783 | Africa | Zambia | 228900054 | 116484694 |
| 3800 | Europe | Russian Federation | 0 | 0 |
| 3819 | Africa | Ethiopia | 394214 | 178710 |
| 3846 | Asia | Indonesia | 0 | 0 |
| 3847 | Asia | Indonesia | 0 | 0 |
| 3859 | Asia | Indonesia | 1674789 | 736907 |
| 3866 | Africa | Congo. Dem. Rep. | 15556555 | 6637463 |
| 3891 | South America | Colombia | 171360833 | 68544333 |
| 3901 | Africa | Liberia | 0 | 0 |
| 3915 | Africa | Ghana | 2686428 | 1253667 |
| 3986 | Africa | Sudan | 3323560178 | 897361248 |
| 4055 | Africa | Senegal | 152902688 | 32619240 |
| 4059 | Africa | Senegal | 69080416 | 15658228 |
| 4059 | Africa | Senegal | 83479768 | 18922081 |
| 4060 | Africa | Zambia | 199875 | 91942 |
| 4117 | Asia | Philippines | 0 | 0 |
| 4171 | South America | Brazil | 63484907 | 37244479 |
| 4218 | South America | Brazil | 73180840 | 19514891 |
| 4219 | South America | Brazil | 9481142 | 3033966 |
| 4287 | Africa | Zambia | 12068665 | 5632044 |
| 4341 | Africa | Ghana | 4422762 | 2063956 |
| 4389 | Africa | Botswana | 206593068 | 71618930 |
| 4389 | Africa | Botswana | 251777196 | 87282761 |
| 4411 | Europe | Romania | 41373404 | 26478979 |
| 4412 | Europe | Romania | 2475607 | 1331326 |
| 4412 | Europe | Romania | 3762710 | 2023502 |
| 4412 | Europe | Romania | 3820684 | 2054679 |
| 4413 | Europe | Romania | 11689316 | 4675727 |

| Deal _ID | Target region | Target country | Scenario 100% cultivated - current irrigation efficiency [m3] | Scenario 100% cultivated - irrigation efficiency 75% [m3] |
|---|---|---|---|---|
| 4414 | Europe | Romania | 19819588 | 6870791 |
| 4415 | Europe | Romania | 1332549 | 657391 |
| 4417 | Europe | Romania | 5635044 | 2779955 |
| 4419 | Europe | Romania | 18447412 | 6395103 |
| 4419 | Europe | Romania | 13398648 | 4644865 |
| 4419 | Europe | Romania | 13398648 | 4644865 |
| 4433 | Africa | Malawi | 33812945 | 27952034 |
| 4433 | Africa | Malawi | 16969416 | 14028050 |
| 4470 | Asia | Indonesia | 0 | 0 |
| 4493 | Africa | Mozambique | 2509002 | 802881 |
| 4503 | Europe | Romania | 8695918 | 4203027 |
| 4509 | Europe | Romania | 7353392 | 4395694 |
| 4509 | Europe | Romania | 11886169 | 7105288 |
| 4509 | Europe | Romania | 10916540 | 6525665 |
| 4513 | Europe | Romania | 4471430 | 1967429 |
| 4513 | Europe | Romania | 10907501 | 4799300 |
| 4513 | Europe | Romania | 5971778 | 2627582 |
| 4527 | Africa | Egypt. Arab Rep. | 389648588 | 145468806 |
| 4557 | Asia | Mongolia | 512661 | 280255 |
| 4563 | Europe | Romania | 66128380 | 37913605 |
| 4563 | Europe | Romania | 32818295 | 18815823 |
| 4582 | Africa | Ghana | 4238026 | 1977746 |
| 4583 | Africa | Ghana | 1581532 | 716961 |
| 4594 | Europe | Ukraine | 1827489 | 1187868 |
| 4594 | Europe | Ukraine | 2047337 | 1330769 |
| 4594 | Europe | Ukraine | 1429260 | 929019 |
| 4616 | Europe | Ukraine | 8325249 | 5272658 |
| 4618 | Europe | Ukraine | 0 | 0 |
| 4618 | Europe | Ukraine | 0 | 0 |
| 4730 | Africa | Ghana | 12608793 | 5884103 |
| 4732 | Europe | Bulgaria | 3976641 | 1590656 |
| 4745 | Africa | Uganda | 24536207 | 12104529 |
| 4802 | Asia | Indonesia | 3447588 | 1516939 |
| 4802 | Asia | Indonesia | 3417159 | 1503550 |
| 4924 | Africa | Zimbabwe | 115861825 | 73379156 |
| 4929 | Africa | Sierra Leone | 53045000 | 14145333 |
| 4930 | Africa | Senegal | 11508490 | 1994805 |
| 4931 | Africa | Senegal | 256762043 | 47928915 |
| 4940 | Africa | Ghana | 17076374 | 7968974 |
| 5024 | Africa | Uganda | 0 | 0 |
| 5316 | Africa | Ghana | 486023 | 149047 |
| 5385 | Europe | Serbia | 0 | 0 |

| Deal _ID | Target region | Target country | Scenario 100% cultivated - current irrigation efficiency [m3] | Scenario 100% cultivated - irrigation efficiency 75% [m3] |
|---|---|---|---|---|
| 5389 | Europe | Ukraine | 4244248 | 2018376 |
| 5389 | Europe | Ukraine | 5925769 | 2818033 |
| 5389 | Europe | Ukraine | 1120766 | 532986 |
| 5389 | Europe | Ukraine | 1885248 | 896540 |
| 5501 | Europe | Russian Federation | 393721748 | 209984932 |
| 5519 | Europe | Ukraine | 5557553 | 4223740 |
| 5551 | Europe | Romania | 23215610 | 13619825 |
| 5585 | Africa | Cameroon | 0 | 0 |
| 5586 | Africa | Cameroon | 0 | 0 |
| 5850 | Asia | Myanmar | 216376213 | 69240388 |
| 5863 | Asia | Myanmar | 0 | 40495093 |
| 6057 | Africa | Ghana | 0 | 0 |
| 6079 | Europe | Ukraine | 21171809 | 12138504 |
| 6079 | Europe | Ukraine | 14572306 | 8354789 |
| 6086 | Europe | Ukraine | 782296 | 531961 |
| 6087 | Europe | Ukraine | 1034716 | 558747 |
| 6096 | Europe | Romania | 0 | 0 |
| 6096 | Europe | Romania | 0 | 0 |
| 6097 | Europe | Romania | 13939546 | 6412191 |
| 6101 | Europe | Russian Federation | 1656875 | 1192950 |
| 6104 | Europe | Ukraine | 967356 | 806130 |
| 6104 | Europe | Ukraine | 1303890 | 1086575 |
| 6113 | Europe | Ukraine | 0 | 0 |
| 6113 | Europe | Ukraine | 7370495 | 6092943 |
| 6114 | Europe | Ukraine | 1424261 | 1144157 |
| 6115 | Europe | Ukraine | 1840407 | 1521403 |
| 6119 | Europe | Ukraine | 0 | 0 |
| 6727 | Asia | Indonesia | 0 | 0 |
| 6777 | Asia | Myanmar | 434370258 | 192777658 |
| **Total** | | | **27708700972** | **10848469241** |

**Supplementary figure S1**

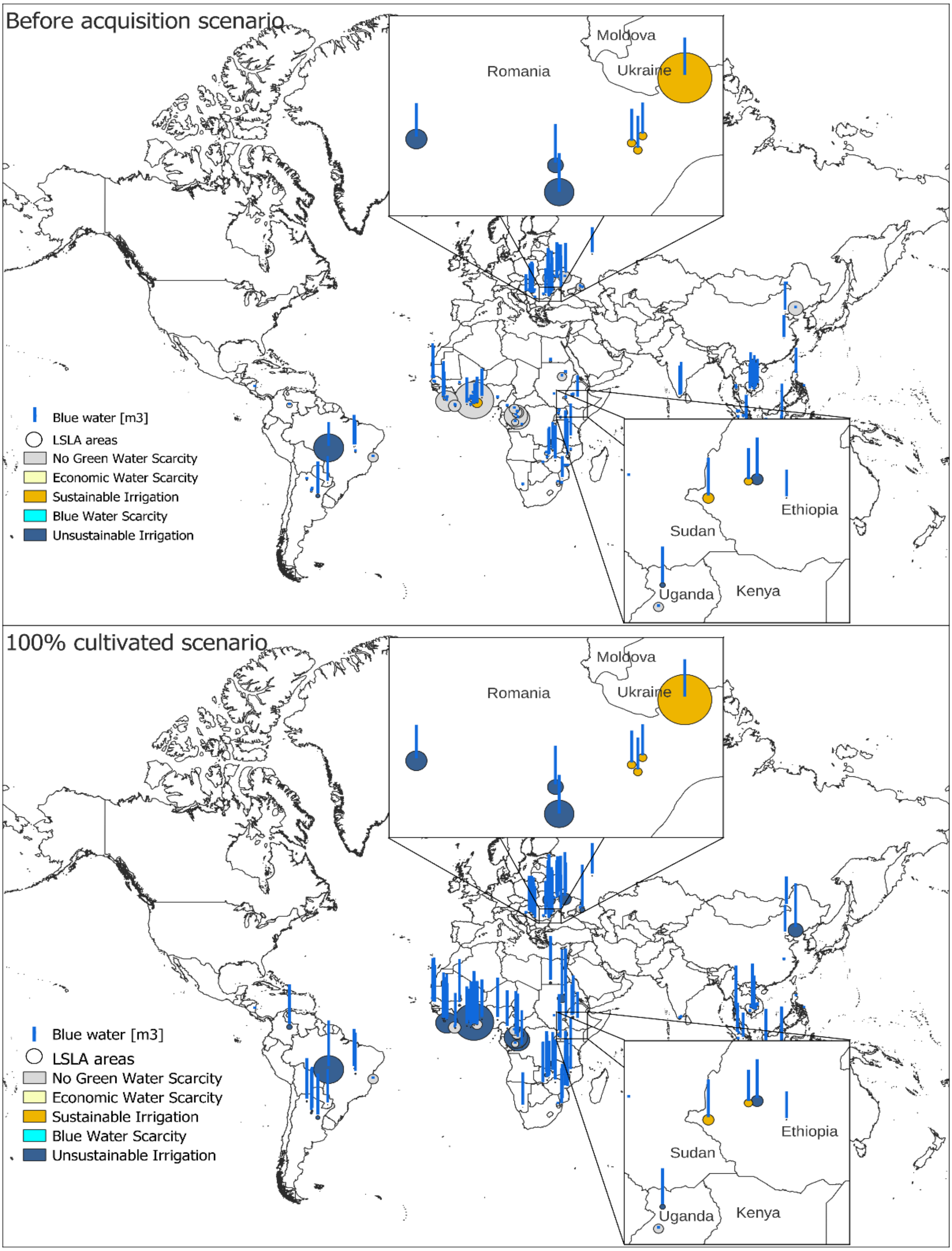


**Fig. S1 Large scale land acquisitions distribution in the before acquisition (top) and 100% cultivation (bottom) scenarios.** The circles are proportional to the acquired areas while the blue bars are proportional to the volume of blue water demand of each deal. Each circle corresponds to a single location of each deal and is colored according to a condition of no green water scarcity (NO GWS). blue water scarcity (BWS) and economic water scarcity (EWS). following the color scheme in Fig. 1 and 2. It is found that only about 47 deals accounting for about 0.2Mha are in a condition of economic water scarcity. while the majority of the land deals suffers from water competition (Blue Water Scarcity).

**Supplementary figure S2**

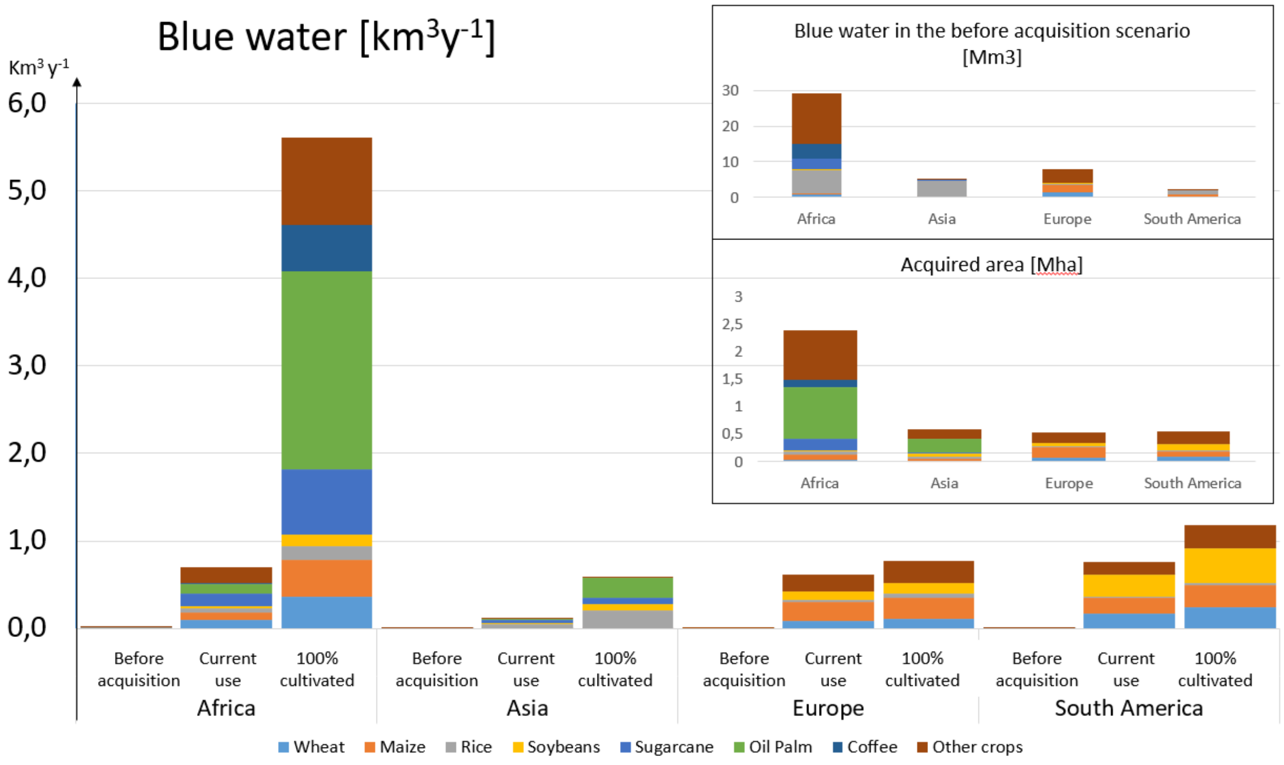


**Fig. S2** Blue water volumes associated with the main crops in the before acquisition scenario. current use scenario and 100% cultivated scenario at complete yield gap closure. Unit: km3/y. Being Africa the most targeted country (62.5% of the acquired area). the demand for additional water is higher than the other region. In the inset on the top right the enlargement of the blue water for the before acquisition scenario for each group and below the area acquired in each target macro region.

**Supplementary figure S3**

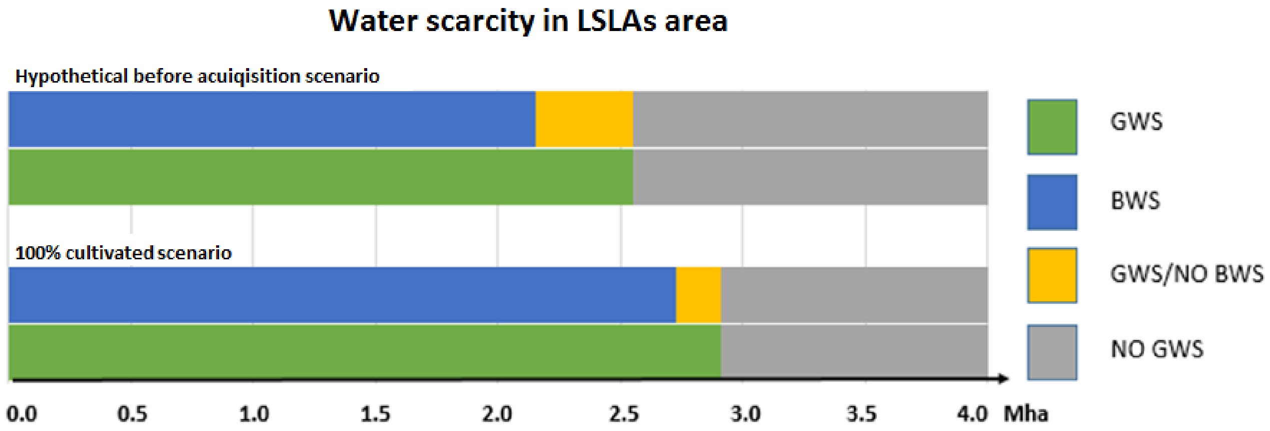


**Fig. S3** Water scarcity under the 100% cultivated scenario and in a hypothetical scenario in which the entire acquired land is cultivated with the crops harvested before the land acquisition. GWS stands for green water scarcity. BWS for blue water scarcity.

**Supplementary figure S4**

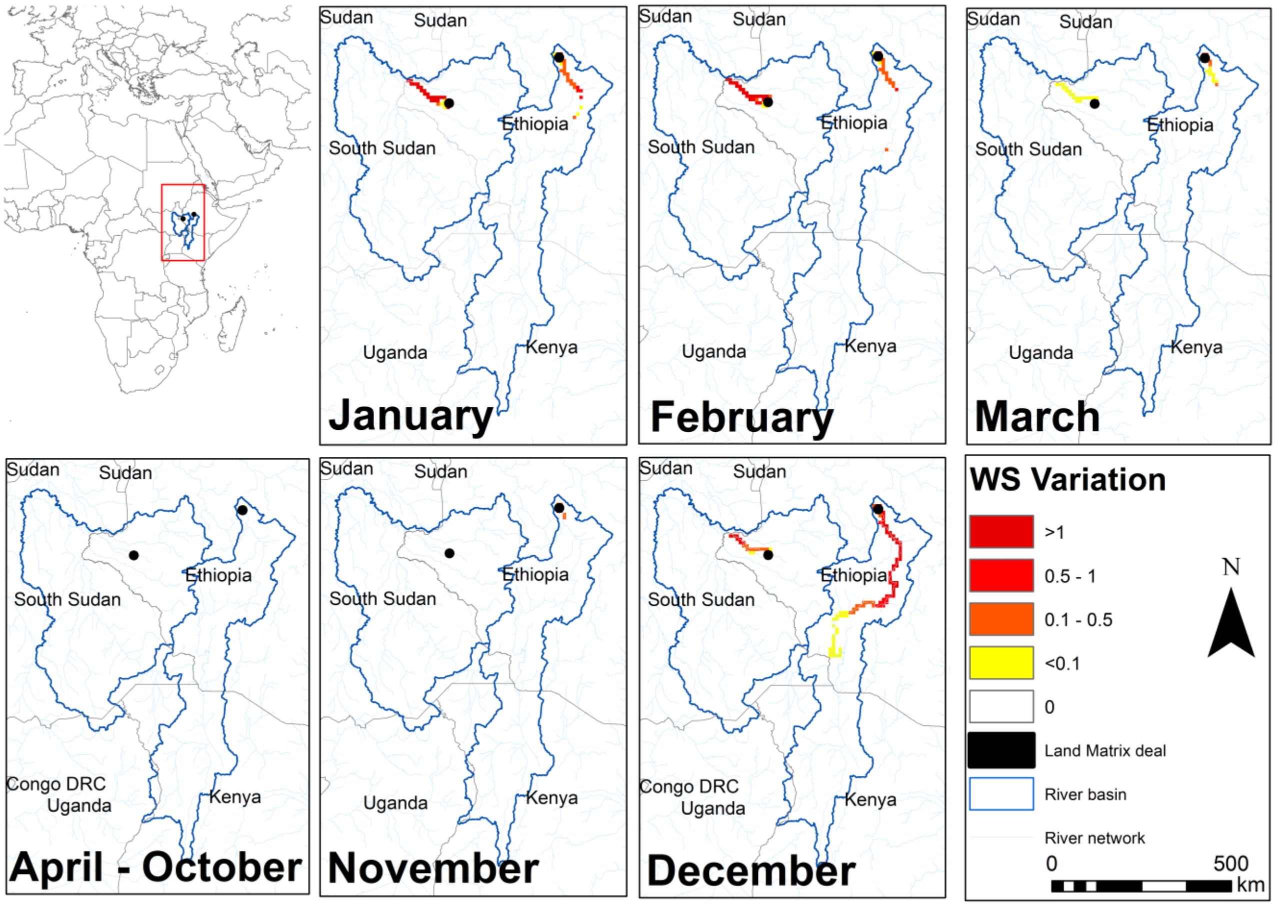


**Fig. S4** Water scarcity variation for the two deals( #1205 and #1244) located in the Oromya region in the Omo River Basin and in the Gambella region in a tributary of Nile River. Both deals have a size of about 15000ha. harvested with maize. sugarcane cotton and sunflower.
